# Hydrophilic PVI-HEA-Based Osmium Redox Polymers for Enhanced Electrochemical Glucose Sensing

**DOI:** 10.3390/bios16080430

**Published:** 2026-08-07

**Authors:** Tae-Won Seo, Won-Yong Jeon, Hyug-Han Kim, Young-Bong Choi

**Affiliations:** 1 Department of Chemistry, College of Science and Technology, Dankook University, Cheonan 31116, Chungcheongnam-do, Republic of Korea; xodnjs7508@dankook.ac.kr; 2 Graduate School of Management of Technology, Hoseo University, 20 Hoseo-ro 79 beon-gil, Baebang-eup, Asan-si 31499, Chungcheongnam-do, Republic of Korea; powerwy@hoseo.edu; 3 Fine Dust & Net Zero Research Institute, Hoseo University, 20 Hoseo-ro 79 beon-gil, Baebang-eup, Asan-si 31499, Chungcheongnam-do, Republic of Korea; 4 Eco Upcycling R&D Center, Hoseo University, 20 Hoseo-ro 79 beon-gil, Baebang-eup, Asan-si 31499, Chungcheongnam-do, Republic of Korea; 5 Department of Cosmedical Materials, College of Bio-Convergence, Dankook University, Cheonan-si 31116, Chungcheongnam-do, Republic of Korea

**Keywords:** osmium redox polymer, glucose sensor, FAD-dependent glucose dehydrogenase, hydrophilic copolymer, continuous glucose monitoring (CGM)

## Abstract

Hydrophilic osmium(Os)-based redox polymers were designed as electron-transfer mediators for fungal flavin adenine dinucleotide-dependent glucose dehydrogenase (FAD-GDH)-based glucose sensors. Poly(vinylimidazole-co-hydroxyethyl acrylate) (PVI-HEA) copolymers with different HEA compositions were synthesized and coordinated with Os(dmo-bpy)_2_Cl_2_ to prepare PVI-HEA-Os(dmo-bpy)_2_Cl_2_ redox mediators. The synthesized mediator systems were characterized using ^1^H-nuclear magnetic resonance spectroscopy, Fourier transform infrared spectroscopy, ultraviolet–visible spectroscopy, field emission scanning electron microscopy/energy dispersive spectroscopy, zeta potential analysis, cyclic voltammetry, and electrochemical impedance spectroscopy. The results confirmed the successful formation of Os redox polymer structures and their immobilization on the electrode surface. The electrochemical behavior and glucose sensing performance strongly depended on the PVI-HEA composition. Among the compositions tested, PVI-HEA(3.5:1)-Os(dmo-bpy)_2_Cl_2_ showed the strongest redox current response, stable aqueous dispersion behavior, and relatively low interfacial charge-transfer resistance. Glucose-sensing measurements using FAD-GDH/mediator-modified electrodes showed linear current responses over the glucose concentration range of 1.25–20 mM. The PVI-HEA(3.5:1)-Os(dmo-bpy)_2_Cl_2_-based electrode showed the highest sensitivity of 16.18 μA cm^−2^ mM^−1^, which was significantly higher than those observed at lower-HEA compositions. The optimized mediator system also showed selective glucose responses against representative biological interferents, including ascorbic acid, uric acid, dopamine, and serotonin. Stable catalytic current responses were maintained under Human Plasma-Like Medium conditions, suggesting improved matrix tolerance compared to conventional PVI-based Os redox polymers. The improved sensing performance was attributed to the hydrophilic polymer environment introduced by the HEA units, which may facilitate favorable interfacial charge-transfer behavior within the enzyme–mediator layer. The results show that the hydrophilic copolymer composition plays an important role in the electrochemical behavior and glucose sensing performance of Os redox polymer mediators. The proposed PVI-HEA-Os(dmo-bpy)_2_Cl_2_ system may be a promising candidate for future enzymatic glucose sensing and continuous glucose monitoring-related applications.

## 1. Introduction

Diabetes management is increasingly moving away from single-point glucose testing toward continuous glucose monitoring (CGM) systems that can track glucose changes over time. Conventional disposable glucose meters are simple and used widely because they provide glucose values from a small blood sample. Nevertheless, these devices only measure glucose at a single point in time and cannot fully capture the rapid fluctuations in glucose caused by meals, exercise, sleep, medication, or metabolic conditions. In particular, sudden hypoglycemia or hyperglycemia may not be detected accurately with intermittent measurements alone. Therefore, there is an increasing demand for glucose-sensing systems that can monitor glucose levels continuously and reliably for long periods [1,2,3,4,5,6,7].

Among various glucose-sensing methods, electrochemical glucose sensors are used most widely in commercial devices because they provide rapid responses, easy miniaturization, and quantitative analysis with a small sample volume. In these systems, glucose oxidation by enzymes produces redox reactions that are converted into electrical current signals. For long-term glucose monitoring systems such as CGM, stable electron transfer between the enzyme, mediator, and electrode is crucial because the sensor performance strongly depends on the interfacial electrochemical behavior [5,8,9,10].

Enzyme-based electrochemical glucose sensors are generally classified according to the mechanism of electron transfer from the enzyme to the electrode. Early glucose oxidase (GOX)-based sensors used dissolved oxygen as an electron acceptor or detected hydrogen peroxide produced during glucose oxidation. Nevertheless, these systems can be affected by the oxygen concentration and usually require relatively high operating potentials, which may interfere with biological compounds such as ascorbic acid or uric acid. To reduce these limitations, glucose dehydrogenase (GDH)-based glucose sensors have been actively studied because they show lower oxygen dependence. Among the different GDH enzymes, fungal flavin adenine dinucleotide-dependent glucose dehydrogenase (FAD-GDH) is particularly well-suited for glucose sensing because of its high glucose selectivity and good thermal and pH stability [11,12,13].

In FAD-GDH-based glucose sensing, electrons generated during glucose oxidation must move from the enzyme redox center to the electrode surface. On the other hand, direct electron transfer is usually difficult because the active site of the enzyme is buried deep within the protein structure. Therefore, electron-transfer mediators are needed to shuttle electrons between the enzyme and the electrode. These mediators help generate a stable catalytic current at low operating potentials and reduce interference from oxidizable biological molecules. In addition, the mediators used for CGM systems should remain stable inside the electrode layer during long-term operation without significant leaching [14,15,16,17,18,19,20].

Potassium hexacyanoferrate, ferrocene derivatives, and ruthenium complexes have been studied widely as second-generation redox mediators for glucose sensors. Although these materials show rapid redox reactions and relatively low redox potentials, they still have limitations for long-term CGM applications. For example, potassium hexacyanoferrate has high water solubility and good electron-transfer ability with FAD-GDH, but it can easily leach from the electrode layer. Ferrocene derivatives enable electrochemical tuning via structural modification, but their long-term stability in aqueous environments is limited. Therefore, new mediator systems that can provide stable electrochemical behavior, strong immobilization, and efficient electron transfer under biological conditions are needed [20,21].

Osmium (Os)-based redox polymers have attracted attention as promising mediator systems for enzyme-based glucose sensors [22,23,24,25,26,27,28]. The redox potential of Os complexes can be adjusted by modifying the ligand structures, and Os complexes can form polymeric mediator systems through coordination with imidazole- or pyridine-containing polymers [23,24,25,26]. Compared with small-molecular mediators, Os redox polymers can remain more stably inside the enzyme layer and provide multiple redox-active sites near the enzyme [24,25,26]. Accordingly, Os redox polymers are considered useful materials for CGM applications requiring long-term electrochemical stability [27,28,29,30,31,32,33].

The sensing performance of Os redox polymers is determined by the redox potential of the Os complex and the physicochemical properties of the polymer matrix. Conventional PVI-based Os redox polymers provide multiple immobilized redox centers and improved mediator retention compared to freely diffusible small-molecule mediators. Nevertheless, the hydration state, ionic accessibility, and interfacial properties of the polymer layer can influence redox-site accessibility, charge compensation, and electron-transfer behavior within the enzyme–mediator interface. Previous studies have focused primarily on controlling the Os coordination structure, redox potential, mediator loading, and crosslinking chemistry. By contrast, systematic modulation of the hydrophilic environment through changes in the PVI-based copolymer composition has attracted less attention [34,35,36,37,38].

In the present design, the vinylimidazole units provide coordination sites for the Os complex, while the HEA units introduce hydroxyl-bearing side chains that are expected to produce a more hydrophilic polymer environment. The effects of the copolymer composition on aqueous dispersion, redox behavior, interfacial charge-transfer characteristics, and glucose-sensing performance can be evaluated systematically by varying the vinylimidazole-to-HEA ratio while maintaining the same Os precursor and electrode-preparation conditions. Therefore, the principal novelty of the proposed PVI-HEA-Os system lies in the composition-dependent control of the polymer microenvironment, which is difficult to achieve using conventional PVI-based Os redox polymers without HEA-derived hydrophilic side chains.

In this study, a hydrophilic Os redox polymer system was designed for FAD-GDH-based glucose sensing. First, an Os(dmo-bpy)_2_Cl_2_ precursor containing 4,4′-dimethoxy-2,2′-bipyridine (dmo-bpy) ligands was synthesized. Poly(vinylimidazole-co-hydroxyethyl acrylate) (PVI-HEA) was used as a polymer ligand to prepare the PVI-HEA-Os(dmo-bpy)_2_Cl_2_ complex. In the present study, PVI-HEA copolymers with different HEA compositions were coordinated with Os(dmo-bpy)_2_Cl_2_ and systematically evaluated as redox mediators for FAD-GDH-based glucose sensing. HEA is used widely in biomaterials because it can form stable aqueous environments around biological molecules [34,35].

This study examined how HEA composition affects the electrochemical behavior and glucose-sensing performance of Os redox polymers. Accordingly, PVI-HEA copolymers with different HEA compositions were synthesized. The resulting Os redox polymers were systematically compared. The effects of the HEA composition on the scan-rate-dependent electrochemical behavior, redox current response, charge-transfer resistance, and glucose catalytic current were analyzed to determine how the hydrophilic polymer composition influences the apparent electron-transfer behavior in the sensing interface [39,40,41,42].

The synthesized PVI-HEA-Os(dmo-bpy)_2_Cl_2_ complexes were characterized using ^1^H- magnetic resonance (NMR) spectroscopy, Fourier transform infrared (FT-IR) spectroscopy, and ultraviolet–visible (UV–Vis) spectroscopy. Their electrochemical properties were investigated by cyclic voltammetry (CV). In addition, field-emission scanning electron microscopy (FE-SEM), energy-dispersive spectroscopy (EDS), zeta potential analysis, and electrochemical impedance spectroscopy (EIS) were used to analyze the surface and interfacial properties. The glucose sensing performance was evaluated using FAD-GDH-modified electrodes at different glucose concentrations, and selectivity tests were conducted in the presence of representative electrochemical interferents, including ascorbic acid, uric acid, dopamine, and serotonin.

This study focused on the relationship between the hydrophilic polymer composition and electrochemical sensing behavior in Os redox polymer systems. By systematically controlling the HEA composition, this study aimed to provide a useful design strategy for developing Os redox polymer mediators with improved glucose-sensing performance, stable electrochemical responses, and resistance to interference for future CGM applications.

## 2. Materials and Methods

### 2.1. Chemicals and Reagents

Ammonium hexachloroosmate(IV), 4,4′-dimethoxy-2,2′-bipyridine (dmo-bpy), 1-vinylimidazole, azobisisobutyronitrile (AIBN), poly(ethylene glycol) diglycidyl ether (PEGDGE), D-(+)-glucose, ascorbic acid (AA), dopamine hydrochloride (DA), uric acid (UA), serotonin (5-HT), and sodium hydrosulfite were purchased from Sigma–Aldrich Co., Ltd. (Milwaukee, WI, USA) and used as received. 2-Hydroxyethyl acrylate (HEA), ethylene glycol, ethyl acetate, diethyl ether, ethanol, and methanol were purchased from Daejung Chemicals & Metals Co., Ltd. (Siheung, Republic of Korea). FAD-dependent glucose dehydrogenase (FAD-GDH) was obtained from Toyobo Co., Ltd. (Osaka, Japan). Human Plasma-Like Medium was purchased from Thermo Fisher Scientific Co., Ltd. (Waltham, MA, USA).

Phosphate-buffered saline (PBS, pH 7.4) was used for the electrochemical measurements. Commercial 1× PBS was used directly, or PBS was prepared using sodium dihydrogen phosphate (NaH_2_PO_4_), disodium hydrogen phosphate (Na_2_HPO_4_), and sodium chloride (NaCl). Potassium ferricyanide (K_3_[Fe(CN)**_6_**]) and potassium ferrocyanide (K_4_[Fe(CN)**_6_**]) were used as redox probes for electrochemical impedance spectroscopy (EIS). All aqueous solutions were prepared using deionized water or Milli-Q-grade water.

A screen-printed carbon electrode (SPCE) was used as the working electrode. The SPCE was prepared by screen-printing carbon conductive ink (Electrodag 423SS, Acheson, Port Huron, MI, USA) onto an OHP film and drying it at room temperature prior to use. The electrochemical measurements were taken using a three-electrode system with the SPCE, platinum wire, and an Ag/AgCl electrode as the working, counter, and reference electrodes, respectively. All chemicals were used as analytical-grade or GR-grade reagents without additional purification.

### 2.2. Equipment for Characterization

The structural, physicochemical, morphological, and electrochemical properties of the synthesized PVI-HEA copolymers and PVI-HEA-Os(dmo-bpy)_2_Cl_2_ redox mediators were analyzed using ^1^H-NMR spectroscopy, FT-IR spectroscopy, UV–Vis spectroscopy, FE-SEM/EDS, zeta potential analysis, electrochemical analysis, and TGA. The ^1^H-NMR spectra were measured using an AVANCE III 400 spectrometer (400 MHz, Bruker, Billerica, MA, USA). The synthesized PVI-HEA copolymers and PVI-HEA-Os(dmo-bpy)_2_Cl_2_ complexes were dissolved in D_2_O or suitable deuterated solvents before analysis.

The FT-IR spectra were obtained using a Spectrum Two FT-IR spectrometer (PerkinElmer, Waltham, MA, USA) in attenuated total reflection (ATR) mode. The measurement range was set to 4000–500 cm^−1^. The UV–Vis absorption spectra were measured using a UV–Vis spectrophotometer (Model 8453, Agilent Technologies, Shanghai, China). The samples were dissolved in deionized water or suitable solvents and analyzed using quartz cuvettes.

The surface morphology and elemental composition of the electrodes were analyzed using FE-SEM/EDS. For sample preparation, PVI-HEA-Os(dmo-bpy)_2_Cl_2_ or enzyme/mediator mixtures were coated onto the surface of SPCEs and dried before analysis. FE-SEM images were used to observe the surface morphology, and EDS analysis was used to confirm the elemental composition on the electrode surface.

The dispersion properties of the synthesized PVI-HEA-Os(dmo-bpy)_2_Cl_2_ complexes were evaluated using a zeta potential analyzer (SZ-100, Horiba, Kyoto, Japan). Each sample was dispersed in deionized water and analyzed at approximately 25 °C. All electrochemical measurements were taken using a CHI 660B electrochemical workstation (CH Instruments, Austin, TX, USA). The thermal stability of the PVI-HEA(3.5:1) copolymer was evaluated by thermogravimetric analysis (TGA N-1500, Sinco, Seoul, Republic of Korea). TGA was performed under an air atmosphere from room temperature to 600 °C at a heating rate of 10 °C/min.

### 2.3. Synthesis of PVI-HEA Copolymer

Poly(vinylimidazole-co-hydroxyethyl acrylate) (PVI-HEA) copolymers were synthesized through the free-radical copolymerization of 1-vinylimidazole and 2-hydroxyethyl acrylate (HEA). The effects of the HEA composition on the electrochemical properties of the redox mediator were examined by adjusting the feed molar ratios of 1-vinylimidazole to HEA to 2:1, 4:1, and 6:1. In this study, PVI-HEA(2:1), PVI-HEA(4:1), and PVI-HEA(6:1) refer to the feed ratios of 1-vinylimidazole to HEA [37,38].

For the synthesis of PVI-HEA(2:1), 1.44 g (0.0118 mol) of 2-hydroxyethyl acrylate and 2.24 g (0.0236 mol) of 1-vinylimidazole were used, and 0.116 g (0.71 mmol) of AIBN was added as an initiator. For PVI-HEA(4:1), 0.74 g (0.0061 mol) of HEA, 2.30 g (0.0244 mol) of 1-vinylimidazole, and 0.098 g (0.60 mmol) of AIBN were used. For PVI-HEA(6:1), 0.56 g (0.0046 mol) of HEA, 2.60 g (0.0276 mol) of 1-vinylimidazole, and 0.106 g (0.65 mmol) of AIBN were used.

All PVI-HEA copolymers were synthesized using the same procedure. First, the required amounts of 2-hydroxyethyl acrylate and 1-vinylimidazole were added to a 100 mL three-neck round-bottom flask, followed by the addition of 50 mL of ethyl acetate. The mixture was stirred at room temperature until the monomers were dissolved. Separately, AIBN was dissolved in 10 mL of ethyl acetate to prepare the initiator solution.

A reflux condenser and dropping funnel were connected to the reaction flask, and the reaction temperature was maintained at 70 °C under a nitrogen atmosphere using an oil bath. The AIBN solution was added slowly through the dropping funnel, and the copolymerization reaction was carried out under reflux conditions for 22 h.

After the reaction, the reaction solution was added dropwise into diethyl ether to precipitate the polymer. The precipitated polymer was collected by vacuum filtration using a 0.45 μm nylon membrane filter and dried in a vacuum oven for 24 h. The dried polymer was redissolved in deionized water and purified using an Amicon stirred cell equipped with a 10 kDa ultrafiltration membrane to remove the unreacted monomers and low-molecular-weight impurities. The purified polymer solution was precipitated again in diethyl ether, filtered through a 0.45 μm nylon membrane filter, and dried in a vacuum oven for 24 h before use.

The actual composition of the synthesized PVI-HEA copolymers was determined from the integral values of the ^1^H-NMR spectra. Based on the calculated composition ratios, the copolymers synthesized at feed ratios of 2:1, 4:1, and 6:1 were designated as PVI-HEA(3.5:1), PVI-HEA(5:1), and PVI-HEA(6.5:1), respectively [43].

### 2.4. Synthesis of Os(dmo-bpy)_2_Cl_2_

Os(dmo-bpy)_2_Cl_2_ was synthesized through a coordination reaction between ammonium hexachloroosmate(IV) and 4,4′-dimethoxy-2,2′-bipyridine (dmo-bpy). First, 0.26 g (0.59 mmol) of ammonium hexachloroosmate(IV) and 0.28 g of dmo-bpy were added to a 100 mL round-bottom flask. Subsequently, 30 mL of ethylene glycol was added as the solvent, and the mixture was stirred at room temperature until well mixed [24,32,33].

A reflux condenser was connected to the reaction flask, and the reaction was carried out under reflux at 180 °C in an oil bath for 30 min. After the reaction, a sodium hydrosulfite solution prepared by dissolving 4.11 g (23.6 mmol) of sodium hydrosulfite in 150 mL of deionized water was added to the reaction mixture. The reduction reaction was then continued for an additional 30 min.

The resulting solid product was collected by vacuum filtration using a 0.45 μm nylon membrane filter. The collected product was dried in an oven at 30 °C for 24 h and used as the Os(dmo-bpy)_2_Cl_2_ precursor.

### 2.5. Synthesis of PVI-HEA-Os(dmo-bpy)_2_Cl_2_

PVI-HEA-Os(dmo-bpy)_2_Cl_2_ redox mediators were prepared through a coordination reaction between the synthesized Os(dmo-bpy)_2_Cl_2_ precursor and PVI-HEA copolymers. As a representative example, PVI-HEA(2:1)-Os(dmo-bpy)_2_Cl_2_ was synthesized as follows [24,32,33]. First, 0.107 g of Os(dmo-bpy)_2_Cl_2_ and 0.078 g of PVI-HEA(2:1) were added to a 100 mL round-bottom flask at an Os:PVI ratio of 1:5 (eq/eq). Ethanol (60 mL) was then added as the reaction solvent. A reflux condenser was attached to the flask, and the reaction mixture was heated to 120 °C in an oil bath for 5 h under reflux.

After the reaction was complete, the reaction solution was precipitated in diethyl ether to obtain the crude product. The resulting precipitate was collected by vacuum filtration using a 0.45 μm nylon membrane filter and dried in an oven at 30 °C for 24 h. The dried product was redissolved in deionized water and purified using an Amicon stirred cell equipped with a 10 kDa ultrafiltration membrane. The purified aqueous solution was reprecipitated in diethyl ether and collected by vacuum filtration. The final product was dried in a vacuum oven for 24 h to obtain the PVI-HEA(2:1)-Os(dmo-bpy)_2_Cl_2_ complex.

PVI-HEA(4:1)-Os(dmo-bpy)_2_Cl_2_ and PVI-HEA(6:1)-Os(dmo-bpy)_2_Cl_2_ were synthesized using the same procedure. Only the composition of the PVI-HEA copolymer was changed, while the Os(dmo-bpy)_2_Cl_2_-to-polymer ratio, solvent, reaction temperature, precipitation, and purification processes were kept constant. Based on the composition ratios calculated from ^1^H-NMR analysis, the final products were designated as PVI-HEA(3.5:1)-Os(dmo-bpy)_2_Cl_2_, PVI-HEA(5:1)-Os(dmo-bpy)_2_Cl_2_, and PVI-HEA(6.5:1)-Os(dmo-bpy)_2_Cl_2_ in the Results and Discussion section.

### 2.6. Preparation of GDH/PVI-HEA-Os(dmo-bpy)_2_Cl_2_/PEGDGE/SPCE

Enzyme electrodes for glucose sensing were prepared by drop-casting a mixed solution containing PVI-HEA-Os(dmo-bpy)_2_Cl_2_ redox mediators, FAD-dependent glucose dehydrogenase (GDH), and poly(ethylene glycol) diglycidyl ether (PEGDGE) onto the surface of the SPCEs [23,26,33,36].

Each PVI-HEA-Os(dmo-bpy)_2_Cl_2_ mediator was dissolved in deionized water at 10 mg/mL to prepare the mediator stock solutions. The crosslinker stock solution was prepared by dissolving FAD-GDH in deionized water at 40 mg/mL and PEGDGE in deionized water at 20 mg/mL.

Immediately before electrode preparation, the casting solution was prepared by mixing the mediator, GDH, and PEGDGE solutions at a 4:4:1 (*v*/*v*/*v*) ratio. Subsequently, 10 μL of the mixed solution was drop-cast onto the working electrode area of the SPCE. After coating, the electrodes were annealed in an oven at 30 °C for 30 min and dried in a desiccator for 24 h. The prepared electrodes were stored in a desiccator or at 4 °C before the electrochemical measurements.

**Scheme 1:**
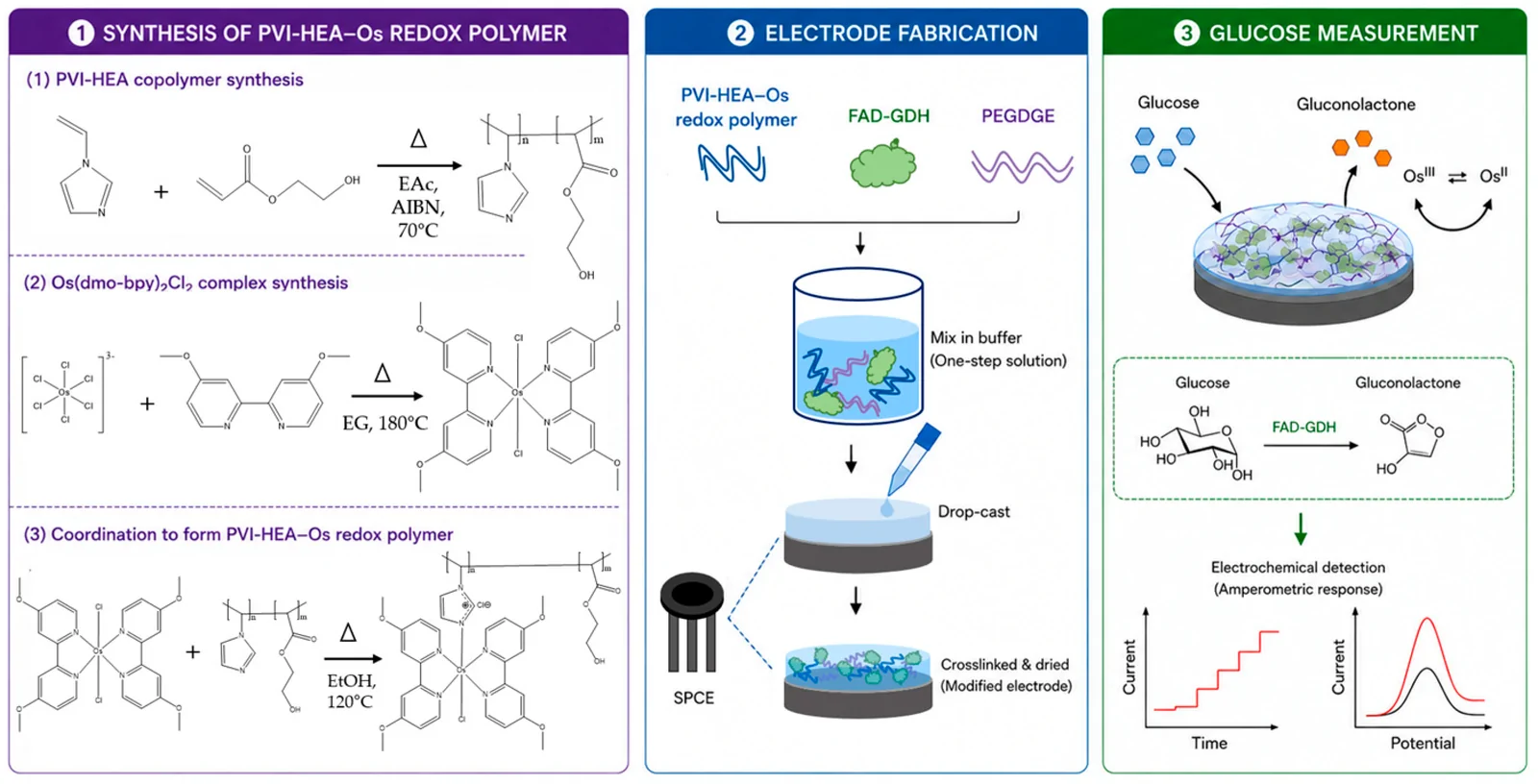
Schematic illustration of the overall experimental workflow for the PVI-HEA(3.5:1)-Os(dmo-bpy)_2_Cl_2_/FAD-GDH/PEGDGE sensor. The scheme shows (**1**) synthesis of the PVI-HEA copolymer, synthesis of the Os(dmo-bpy)_2_Cl_2_ complex, and coordination to form the PVI-HEA(3.5:1)-Os(dmo-bpy)_2_Cl_2_ redox polymer; (**2**) fabrication of the enzyme electrode by mixing the PVI-HEA(3.5:1)-Os(dmo-bpy)_2_Cl_2_ redox polymer with FAD-GDH and PEGDGE, followed by drop-casting onto an SPCE and crosslinking; and (**3**) electrochemical glucose sensing based on FAD-GDH-catalyzed glucose oxidation and mediated electron transfer through the Os redox polymer.

PVI-HEA(3.5:1)-Os(dmo-bpy)_2_Cl_2_/GDH/PEGDGE/SPCE, PVI-HEA(5:1)-Os(dmo-bpy)_2_Cl_2_/GDH/PEGDGE/SPCE, and PVI-HEA(6.5:1)-Os(dmo-bpy)_2_Cl_2_/GDH/PEGDGE/SPCEs were prepared using the same procedure to compare the glucose sensing performance according to the PVI-HEA composition. A schematic overview of the overall experimental procedure is presented in Scheme 1. 

### 2.7. Electrochemical Characterization

CV and EIS were used to examine the electrochemical properties of the PVI-HEA-Os(dmo-bpy)_2_Cl_2_ redox mediators. All electrochemical measurements were carried out using a three-electrode system. A SPCE (diameter 4.0 mm), an Ag/AgCl electrode, and a platinum wire (0.5 mm diameter) were used as the working, reference, and counter electrodes, respectively. Unless stated otherwise, all measurements were taken at room temperature under ambient conditions.

The redox behavior of PVI-HEA-Os(dmo-bpy)_2_Cl_2_ was investigated by CV in 1× PBS (pH 7.4). The basic CV measurements were taken over a potential range from –0.6 V to +0.6 V versus the Ag/AgCl reference electrode.

Scan-rate-dependent CV analysis was performed to compare the electrochemical behavior of mediators with different HEA compositions. The measurements were conducted over a potential range from –1.0 V to +1.0 V, and the scan rate was varied from 0.01 to 2.0 V s^−1^. The oxidation and reduction peak currents obtained at each scan rate were recorded, and the current density was plotted as a function of the scan rate or the square root of the scan rate.

The interfacial charge-transfer properties of the electrodes were analyzed using EIS. EIS measurements were taken in 0.5 M KCl solution containing 2.0 mM K_3_[Fe(CN)_6_]/K_4_[Fe(CN)_6_] as a redox probe. The applied potential was set to 0.266 V versus the Ag/AgCl reference electrode. The AC amplitude was 5 mV, and the frequency range was set from 1 Hz to 100 kHz. The measured impedance spectra are presented as Nyquist plots [40].

### 2.8. Glucose Sensing Measurements

The glucose-sensing performance of the PVI-HEA-Os(dmo-bpy)_2_Cl_2_/GDH/PEGDGE/SPCEs was evaluated using CV and multi-potential step (MPS) measurements. Standard glucose solutions were prepared by dissolving D-(+)-glucose in 1× PBS (pH 7.4). The glucose concentrations used for the measurements were 1.25, 2.5, 5, 10, and 20 mM. Each glucose solution was prepared immediately before the measurement or allowed to stabilize sufficiently before use.

For the electrochemical measurements, 40 μL of glucose solution was dropped onto the working electrode area of the prepared electrode. CV recorded the changes in the catalytic current according to the glucose concentration.

MPS measurements were taken at applied potentials of 0.1, 0.2, and 0.3 V versus the Ag/AgCl reference electrode. Calibration curves were prepared using the current density values obtained from the MPS measurements. The current density measured at 0.2 V was used to compare the glucose-sensing performance according to the PVI-HEA composition.

The same measurement conditions were applied to PVI-HEA(3.5:1)-Os(dmo-bpy)_2_Cl_2_/GDH/PEGDGE/SPCE, PVI-HEA(5:1)-Os(dmo-bpy)_2_Cl_2_/GDH/PEGDGE/SPCE, and PVI-HEA(6.5:1)-Os(dmo-bpy)_2_Cl_2_/GDH/PEGDGE/SPCEs to compare the glucose-sensing performance of the different PVI-HEA compositions.

Amperometric current–time measurements used for low-concentration calibration, repeatability, reproducibility, and continuous operational-stability evaluation were performed at a constant applied potential of 0.2 V versus Ag/AgCl. The detailed experimental procedures for each assessment are described in Section 2.10 and Section 2.11.

### 2.9. Selectivity and Plasma-Like Medium Tests

Representative electrochemical interferents commonly found in biological fluids, including ascorbic acid (AA), uric acid (UA), dopamine (DA), and serotonin (5-HT), were tested to evaluate the selectivity of the PVI-HEA-Os(dmo-bpy)_2_Cl_2_-based glucose sensor. Each interferent solution was prepared at 0.1 mM in 1× PBS (pH 7.4). For comparison, 0.1 mM glucose solutions were also prepared [5,8,9,10,21,44].

Selectivity measurements were carried out using PVI-HEA-Os(dmo-bpy)_2_Cl_2_/GDH/PEGDGE/SPCEs. A 40 μL aliquot of each solution was dropped onto the working electrode area, and the electrochemical measurements were performed under the same MPS conditions used for the glucose-sensing measurements described in Section 2.8. The selectivity was evaluated by comparing the current densities measured at 0.2 V.

Glucose-sensing measurements were also taken in Human Plasma-Like Medium (HPLM) to evaluate electrochemical responses under biologically relevant conditions. Glucose was added to the HPLM, and CV or MPS measurements were taken using the same electrode configuration and experimental conditions. The current responses obtained in PBS and HPLM were compared to assess the signal changes under different matrix conditions [45].

### 2.10. Aqueous Storage Stability

The aqueous storage stability of the optimized PVI-HEA(3.5:1)-Os(dmo-bpy)_2_Cl_2_/FAD-GDH/PEGDGE/SPCE was evaluated over six days using three independently prepared electrodes. After the initial measurement on day 0, the electrodes were immersed in 1× PBS for storage between measurements. The current response of each electrode was measured once daily under identical glucose-sensing conditions using 5.5 mM glucose at 0.2 V versus Ag/AgCl.

The current response measured on day 0 was normalized to 100%, and the relative current response at each subsequent time point was calculated with respect to the initial value. The results are presented as the mean ± standard deviation obtained from three independently prepared electrodes (*n* = 3).

### 2.11. Analytical Performance, Repeatability, and Electrode-to-Electrode Reproducibility

The analytical performance of the optimized PVI-HEA(3.5:1)-Os(dmo-bpy)_2_Cl_2_/FAD-GDH/PEGDGE-modified glucose sensor was evaluated using amperometric current–time measurements at an applied potential of 0.2 V versus Ag/AgCl. Four independently prepared electrodes were immersed separately in 5 mL of 1× PBS. A 1.25 M glucose stock solution prepared in 1× PBS was added in 2 μL aliquots at 60 s intervals, resulting in cumulative glucose concentrations of 0.5, 1.0, 1.5, 2.0, and 2.5 mM.

Electrode-to-electrode reproducibility was evaluated by comparing the amperometric responses obtained from the four independently fabricated electrodes. The low-concentration calibration curve was constructed using the current-density increments obtained at glucose concentrations of 0.5–1.5 mM. Linear regression was used to determine the calibration slope and coefficient of determination.

The limit of detection (LOD) and limit of quantification (LOQ) were estimated according to the ICH Q2(R2) approach using the following equations:LOD = 3.3σ/S, LOQ = 10σ/S where σ is the standard deviation of the background current density obtained from four independently prepared electrodes and S is the slope of the low-concentration calibration curve.

Repeatability was evaluated by performing four consecutive amperometric measurements using the same electrode in glucose-free 1× PBS and in 1× PBS containing 5.5 mM glucose at 0.2 V versus Ag/AgCl. The mean current density, standard deviation, and relative standard deviation (%RSD) were calculated for each glucose condition.

Because glucose was introduced manually without controlled convective mixing, the observed current transient included both the electrode response and solution-mixing and mass-transport contributions. Therefore, an intrinsic numerical response time was not calculated from these measurements.

### 2.12. Operational Stability and Electrochemical Mediator-Retention Test

The operational stability of the optimized glucose sensor was evaluated by continuous amperometric measurement in 1× PBS containing 5.5 mM glucose. The current response was recorded continuously for 12 h at an applied potential of 0.2 V versus Ag/AgCl under static batch conditions without glucose replenishment. The current density measured after the initial transient period was used as the post-transient reference response, and the current retention after 12 h was calculated relative to this value.

The electrochemical retention stability of the immobilized PVI-HEA(3.5:1)-Os(dmo-bpy)_2_Cl_2_ mediator was evaluated by repeated cyclic voltammetric cycling. A total of 250 consecutive CV cycles were recorded in 1× PBS over a potential range of −0.3 to +0.3 V versus Ag/AgCl at a scan rate of 20 mV s^−1^. The voltammograms obtained during the initial and 250th cycles were compared in terms of the Os redox-peak potentials and current densities.

This repeated-cycling experiment was used as an electrochemical assessment of mediator retention. Because the amount of Os released into the electrolyte was not directly quantified, the experiment was not considered a direct quantitative mediator-leaching assay.

## 3. Results

### 3.1. Structural Characterization of PVI-HEA Copolymers

**Figure 1:**
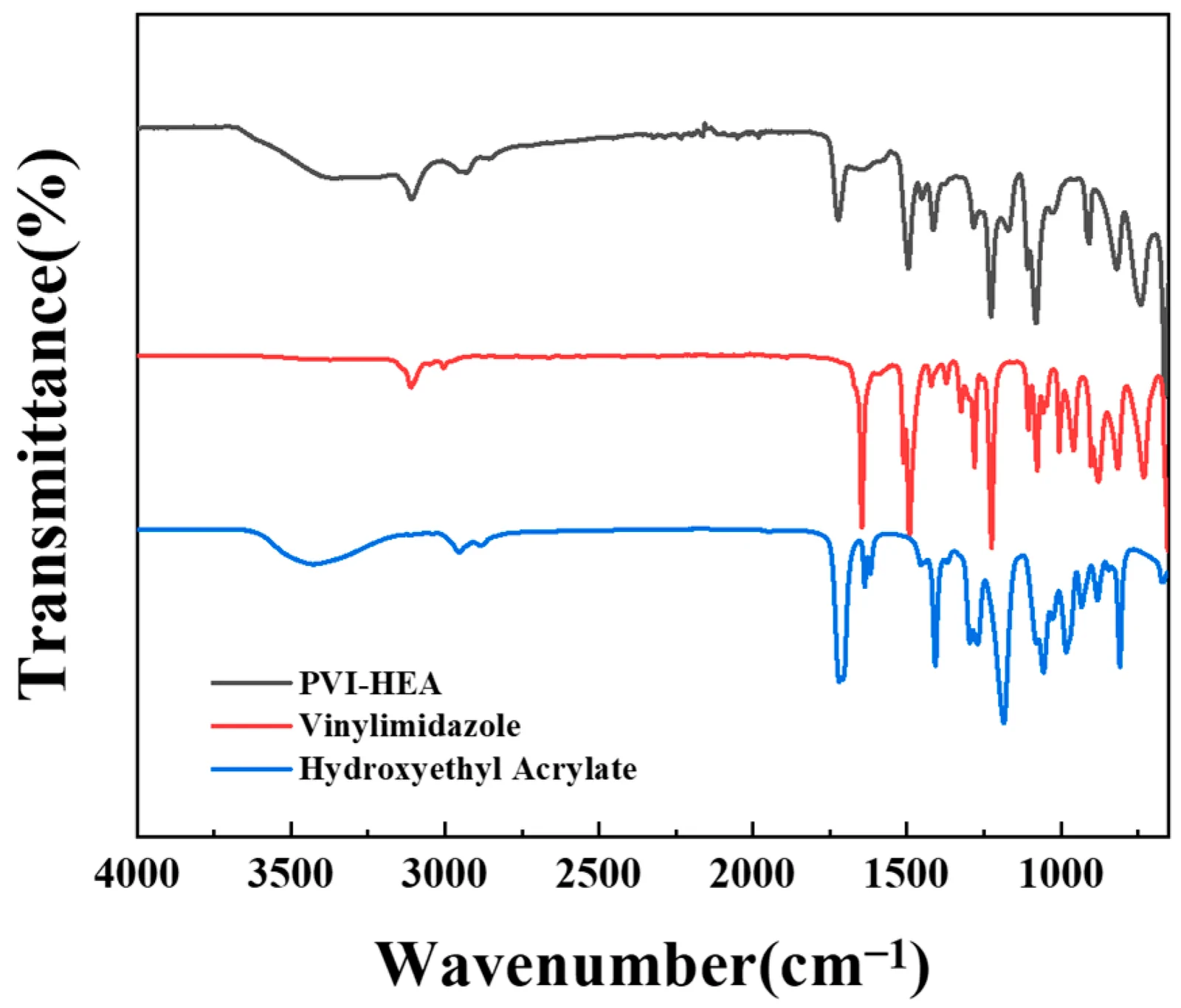
FT-IR spectra of the synthesized PVI-HEA copolymer, 1-vinylimidazole, and 2-hydroxyethyl acrylate measured in ATR mode over the range of 4000–500 cm^−1^. The spectra of PVI-HEA, 1-vinylimidazole, and 2-hydroxyethyl acrylate are shown in black, red, and blue, respectively.

The successful synthesis of the PVI-HEA copolymer was confirmed by comparing the FT-IR spectra of 1-vinylimidazole, 2-hydroxyethyl acrylate (HEA), and the synthesized PVI-HEA, as shown in Figure 1. In the HEA spectrum, a broad O–H stretching band was observed at approximately 3400 cm^−1^, which was assigned to the hydroxyethyl group. In addition, a strong C=O stretching band corresponding to the ester carbonyl group was observed at approximately 1730 cm^−1^. The C–O stretching vibration of the ester group was also detected in the range of 1000–1300 cm^−1^.

The spectrum of 1-vinylimidazole showed the characteristic peaks for the imidazole ring and vinyl group. In particular, the =C–H stretching vibration near 3000 cm^−1^ and the C=C stretching vibration at approximately 1600 cm^−1^ are typical features of the vinylimidazole monomer structure. In the spectrum of the synthesized PVI-HEA copolymer, the O–H stretching band and ester C=O stretching band originating from HEA were observed. At the same time, the peaks associated with the imidazole ring from the vinylimidazole unit were also present. On the other hand, the characteristic vinyl group peaks observed in the monomers became significantly weaker after copolymerization. This result suggests that the vinyl groups of 1-vinylimidazole and HEA participated in the free-radical polymerization reaction to form the polymer backbone.

These FT-IR results suggest that 1-vinylimidazole and HEA were copolymerized to form the PVI-HEA copolymer. In particular, the simultaneous presence of hydroxyl and ester functional groups from HEA together with imidazole-related vibrations from PVI supports the formation of a hydrophilic imidazole-acrylate copolymer rather than a simple PVI structure. The hydrophilic functional groups introduced by HEA may contribute to the formation of a hydrated polymer environment in the Os-based redox polymer system [34,35,36]. They may influence the dispersion behavior and interfacial compatibility of the mediator in enzyme-based glucose sensing systems [37,38].

**Figure 2:**
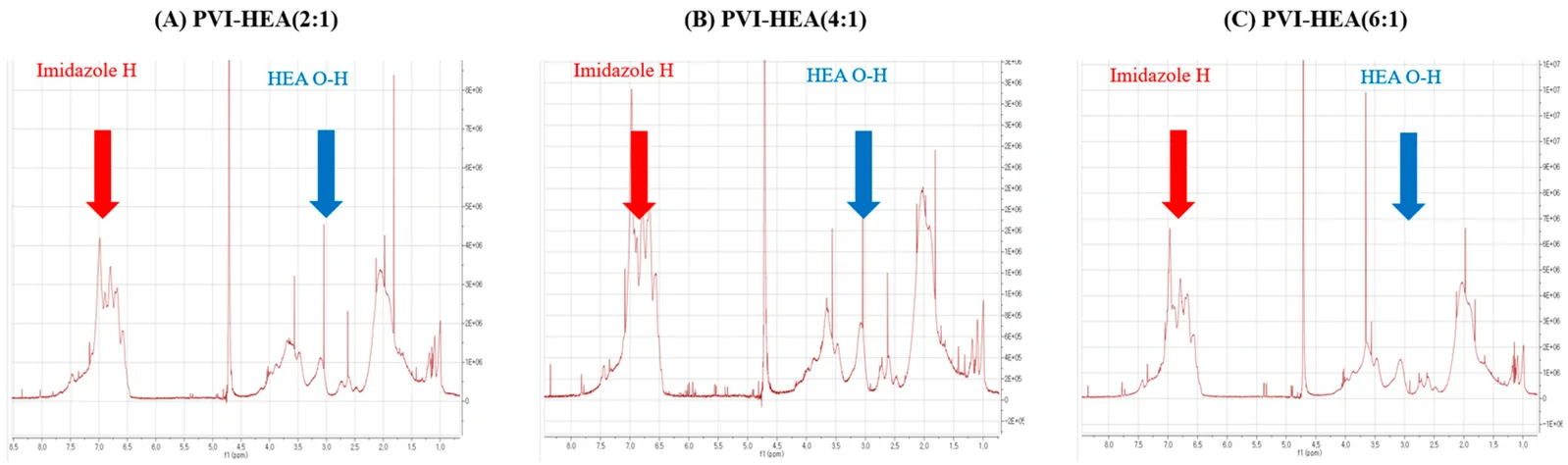
^1^H-NMR spectra of PVI-HEA copolymers synthesized using vinylimidazole-to-HEA feed ratios of 2:1, 4:1, and 6:1. The actual copolymer composition ratios determined from the integrated imidazole- and HEA-derived proton signals were 3.5:1, 5:1, and 6.5:1, respectively.

The composition of the synthesized PVI-HEA copolymers was confirmed by ^1^H-NMR spectroscopy. Figure 2 shows the ^1^H-NMR spectra of PVI-HEA copolymers prepared with different feed ratios. In all PVI-HEA copolymers, proton signals corresponding to the imidazole The composition of the synthesized PVI-HEA copolymers was confirmed by ^1^H-NMR spectroscopy. Figure 2 presents the ^1^H-NMR spectra of PVI-HEA copolymers prepared with different feed ratios. In all PVI-HEA copolymers, proton signals corresponding to the imidazole ring of the 1-vinylimidazole unit were observed at approximately 6.5–7.5 ppm. In addition, proton signals originating from the hydroxyethyl group of the HEA unit were detected in the range of approximately 3.5–4.5 ppm. The broad peaks observed at approximately 1.0–2.5 ppm were assigned to the aliphatic protons of the polymer backbone [43].

The actual compositions of the copolymers were calculated by comparing the integral values of the imidazole ring proton signals and the HEA-related proton signals. The copolymers synthesized with feed ratios of 2:1, 4:1, and 6:1 were determined to have actual composition ratios of PVI-HEA(3.5:1), PVI-HEA(5:1), and PVI-HEA(6.5:1), respectively, based on the ^1^H-NMR results.

Therefore, the relative HEA content in the copolymer decreased as the 1-vinylimidazole feed ratio increased, confirming the successful preparation of PVI-HEA copolymer series with different HEA compositions. Among the synthesized copolymers, PVI-HEA(3.5:1) contained the highest relative amount of HEA, whereas PVI-HEA(6.5:1) showed the lowest HEA content.

These three copolymer compositions were used in the following electrochemical studies to compare the effects of the HEA composition on the redox behavior and glucose-sensing performance of PVI-HEA-Os(dmo-bpy)_2_Cl_2_ redox mediators.

**Figure 3:**
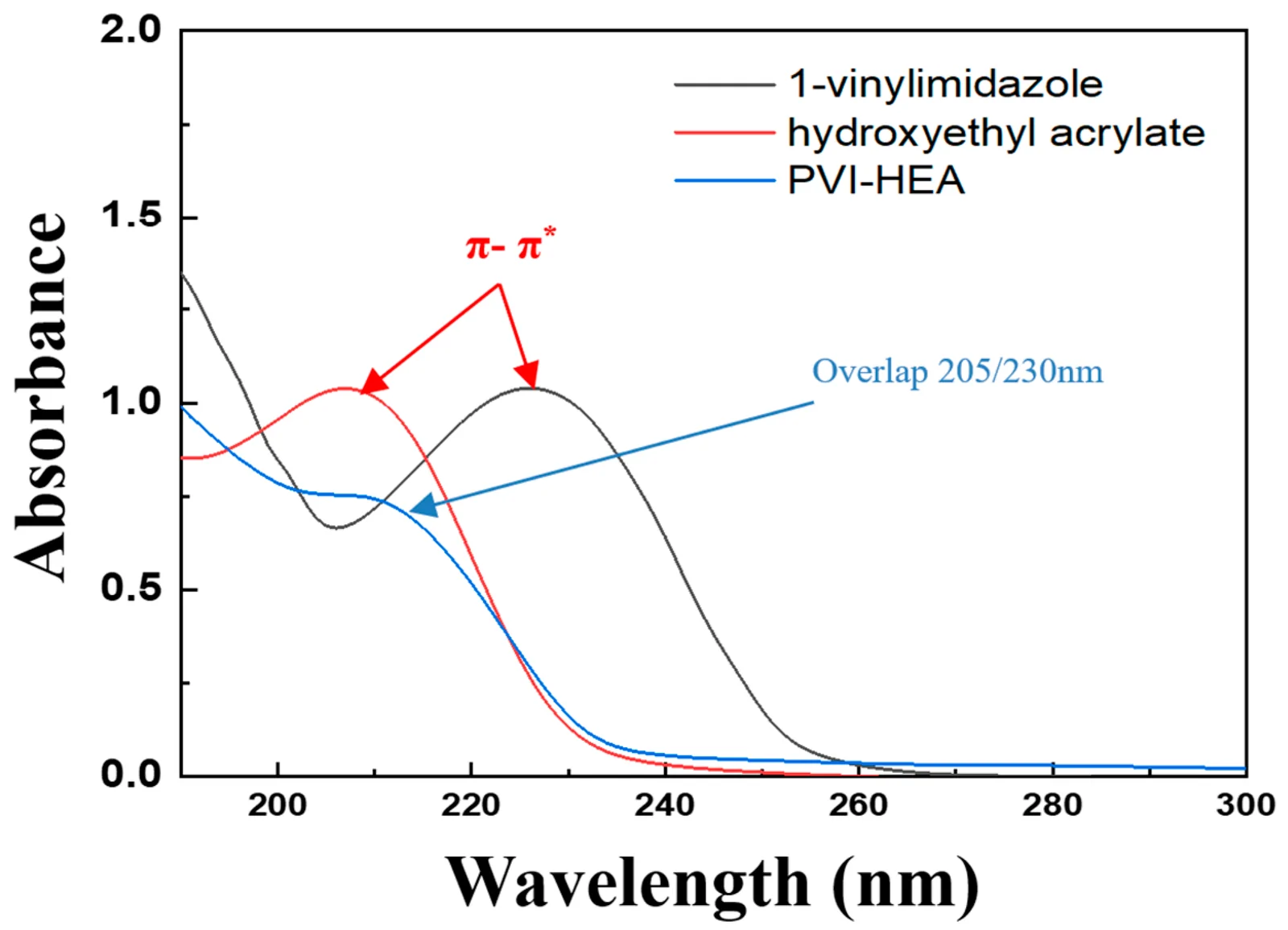
UV–Vis absorption spectra of 1-vinylimidazole, 2-hydroxyethyl acrylate, and the synthesized PVI-HEA copolymer. The spectra of 1-vinylimidazole, HEA, and PVI-HEA are shown in black, red, and blue, respectively.

The formation of the synthesized PVI-HEA copolymer was confirmed by UV–Vis spectroscopy. Figure 3 presents the UV–Vis absorption spectra of 1-vinylimidazole, 2-hydroxyethyl acrylate (HEA), and the synthesized PVI-HEA copolymer.

The 1-vinylimidazole monomer showed a characteristic absorption band near 230 nm, which was assigned to the π–π* transition of the imidazole ring and vinyl group. In the case of HEA, a strong absorption band was observed at approximately 205 nm because of the electronic transitions associated with the acrylate vinyl group and ester carbonyl structure.

In contrast, the UV–Vis spectrum of the synthesized PVI-HEA copolymer showed broader and weaker absorption features than those of the individual monomers. The sharp absorption bands observed in the monomer spectra were reduced significantly after polymerization. This change was attributed to the consumption of vinyl C=C bonds during free-radical copolymerization, in which the vinyl groups of 1-vinylimidazole and HEA participated in forming the polymer backbone.

In particular, the characteristic absorption bands associated with the vinyl groups became less distinct after copolymer formation, suggesting successful polymerization of the monomers. The remaining absorption observed in the PVI-HEA spectrum was attributed mainly to the imidazole ring and ester carbonyl groups retained in the copolymer structure.

These UV–Vis results, together with FT-IR and ^1^H-NMR analyses, showed that 1-vinylimidazole and HEA did not remain as a simple physical mixture but had been successfully copolymerized to form the PVI-HEA copolymer.

**Figure 4:**
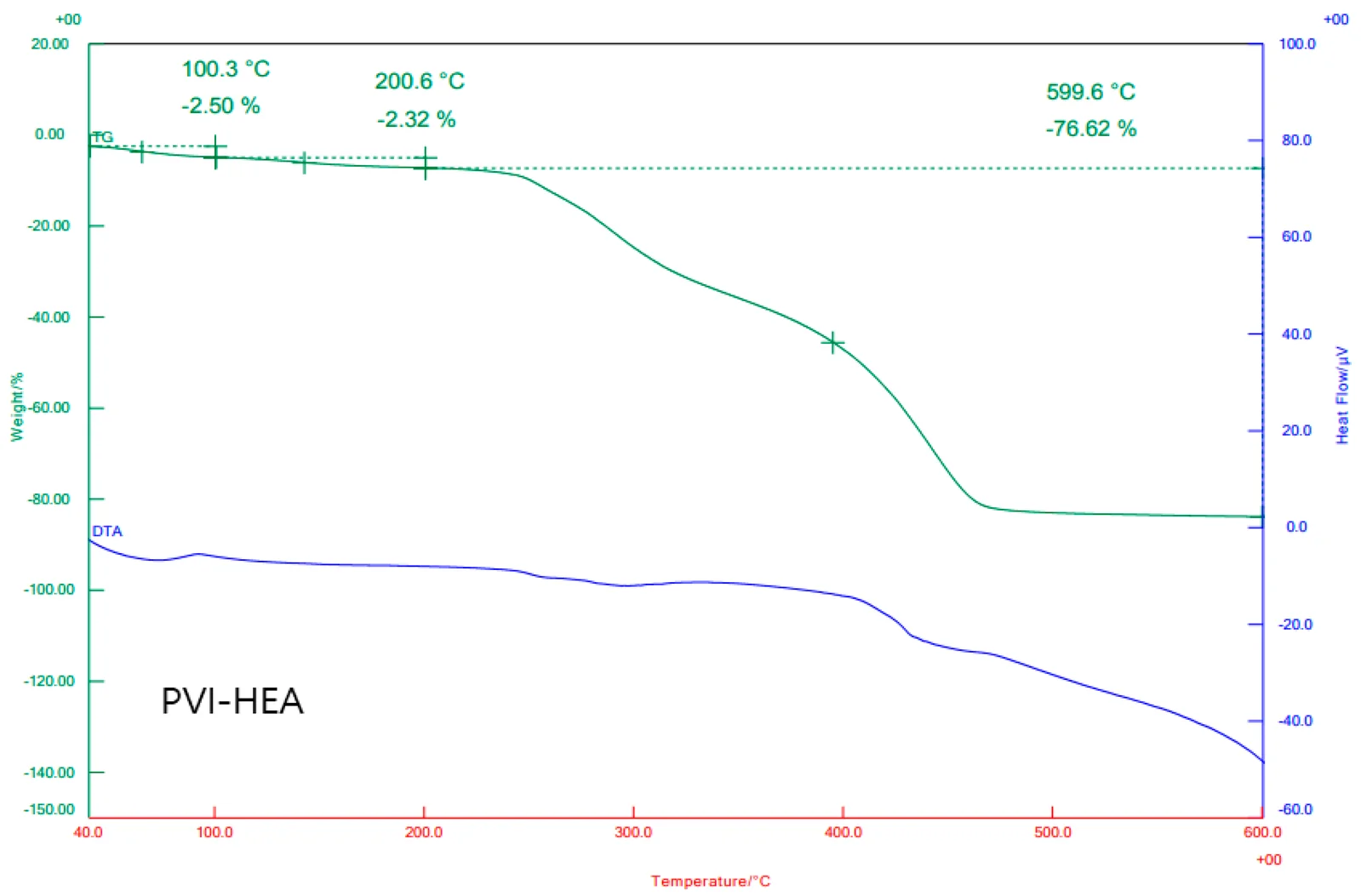
Thermogravimetric and differential thermal analysis curves of the PVI-HEA(3.5:1) copolymer recorded under an air atmosphere from room temperature to 600 °C at a heating rate of 10 °C min^−1^.

The thermal stability and decomposition behavior of the PVI-HEA copolymer were evaluated by TGA. PVI-HEA(3.5:1) was selected as the representative sample for thermal analysis because this copolymer showed the highest glucose-sensing performance among the tested compositions. Figure 4 shows the TGA and DTA curves of PVI-HEA(3.5:1) measured in air at a heating rate of 10 °C/min. The PVI-HEA(3.5:1) copolymer showed only a small initial weight loss below 200 °C. The weight loss was 2.50% at 100.3 °C and remained at 2.32% at 200.6 °C. This initial weight loss was assigned to the removal of physically adsorbed moisture, residual solvent, and weakly bound low-molecular-weight species. The limited mass loss in this region shows that PVI-HEA(3.5:1) maintains good thermal stability below 200 °C. Major thermal decomposition began above approximately 250 °C and proceeded through two main degradation stages. The first major decomposition stage occurred in the range of approximately 250–400 °C. This stage corresponds to the degradation of HEA-derived ester side chains, hydroxyethyl groups, and acrylate segments. Dehydration, ester cleavage, and side-chain decomposition occur predominantly in this temperature region because HEA contains hydroxyl and ester functional groups. The second major decomposition stage appeared more sharply in the range of approximately 400–480 °C. This stage corresponds to thermal breakdown of the main polymer backbone, including the imidazole-containing PVI segments, followed by carbonization. Therefore, the TGA profile confirmed that PVI-HEA(3.5:1) undergoes multi-step thermal degradation, in which HEA-derived side-chain decomposition occurs first, followed by decomposition of the imidazole-containing polymer backbone. At 599.6 °C, the total weight loss reached 76.62%, leaving approximately 23% residual mass. This residual mass was attributed to carbonaceous residues and thermally stable decomposition products formed after polymer degradation in air. Overall, PVI-HEA(3.5:1) possesses sufficient thermal stability under the electrode fabrication and glucose-sensing conditions used in this study, including 30 °C drying and aqueous electrochemical measurements [46,47].

### 3.2. Synthesis and Spectroscopic Characterization of PVI-HEA-Os(dmo-bpy)_2_Cl_2_

**Figure 5:**
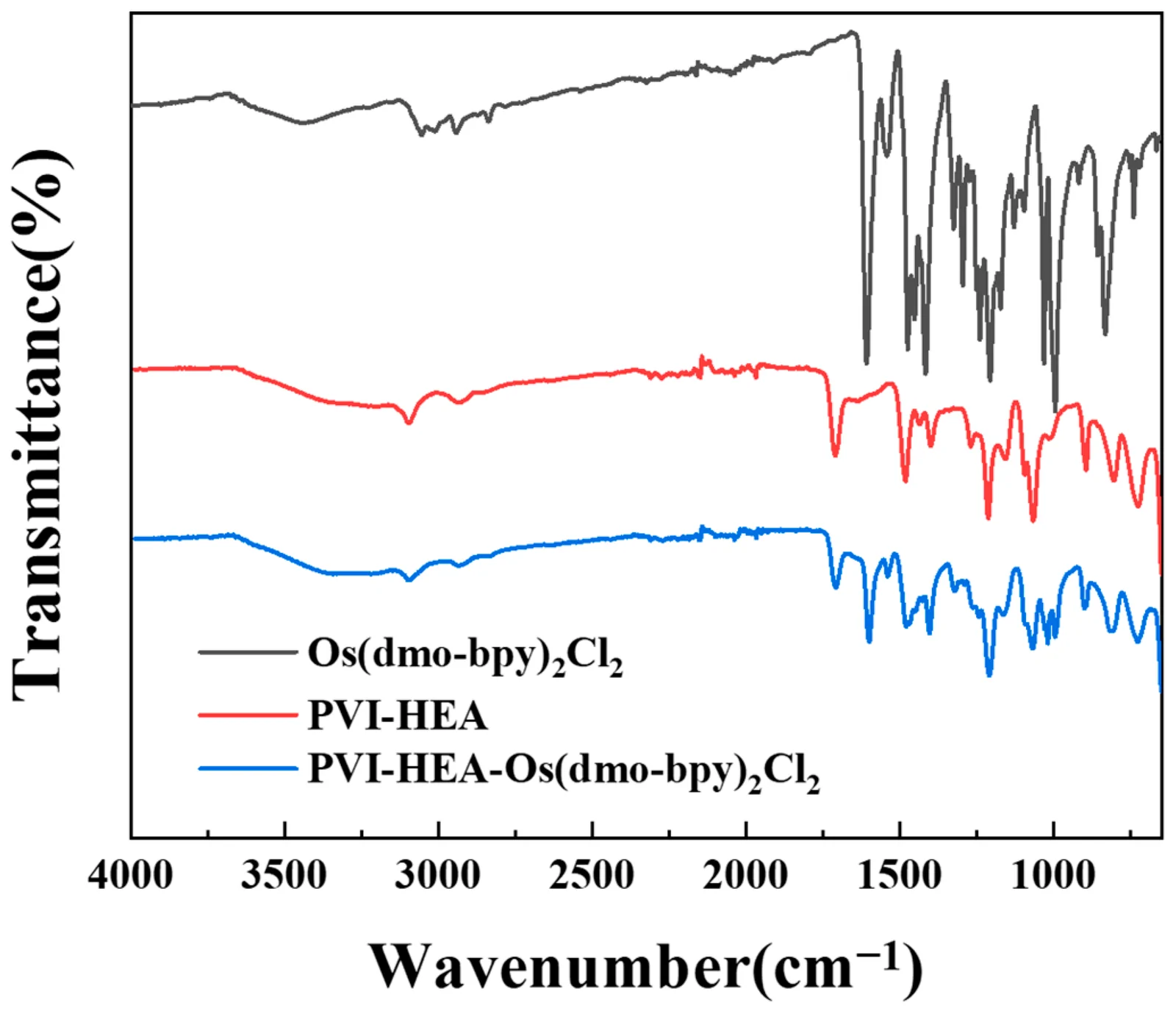
FT-IR spectra of the Os(dmo-bpy)_2_Cl_2_ precursor, PVI-HEA copolymer, and PVI-HEA-Os(dmo-bpy)_2_Cl_2_ redox polymer measured in ATR mode over the range of 4000–500 cm^−1^. The corresponding spectra are shown in black, red, and blue, respectively.

The formation of the PVI-HEA-Os(dmo-bpy)_2_Cl_2_ redox mediator was confirmed by comparing the FT-IR spectra of the Os(dmo-bpy)_2_Cl_2_ precursor, PVI-HEA copolymer, and the final PVI-HEA-Os(dmo-bpy)_2_Cl_2_ complex, as shown in Figure 5.

The spectrum of the Os(dmo-bpy)_2_Cl_2_ precursor revealed the characteristic peaks related to the aromatic ring vibration of the dmo-bpy ligand at approximately 1600 cm^−1^. In addition, peaks associated with the methoxy-substituted bipyridine structure, including C–O stretching and ring vibration bands, were clearly observed in the range of 1300–900 cm^−1^.

The PVI-HEA copolymer showed a broad O–H stretching band at approximately 3400 cm^−1^, which originated from the hydroxyl groups of the HEA units. A strong ester C=O stretching band was also observed near 1730 cm^−1^.

The FT-IR spectrum of the final PVI-HEA-Os(dmo-bpy)_2_Cl_2_ complex revealed characteristic O–H stretching and ester C=O stretching bands from the PVI-HEA copolymer. At the same time, peaks originating from the bipyridine ring and methoxy groups of the Os(dmo-bpy)_2_Cl_2_ precursor were also present in the range of 1600–900 cm^−1^. These results suggest that the PVI-HEA polymer structure and the dmo-bpy-containing Os complex had been incorporated into the final redox mediator structure.

Slight changes in the intensity and shape of several peaks were observed after complex formation compared to the spectrum of the pure PVI-HEA copolymer. These changes are likely associated with coordination interactions between the imidazole groups of PVI-HEA and the Os center. Therefore, the FT-IR results support the successful formation of the PVI-HEA-Os(dmo-bpy)_2_Cl_2_ redox mediator through coordination between the PVI-HEA copolymer and the Os precursor.

**Figure 6:**
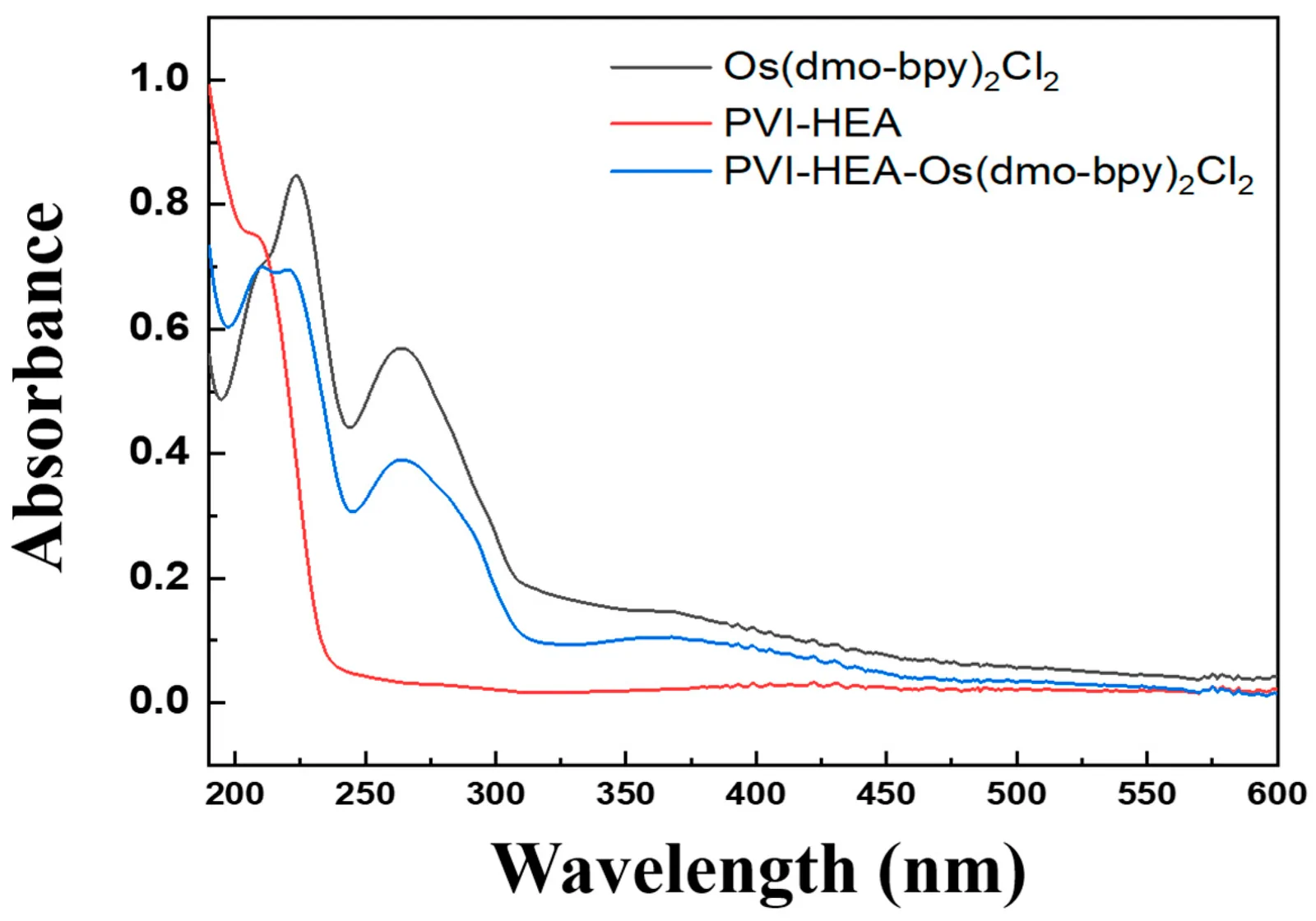
UV–Vis absorption spectra of the Os(dmo-bpy)_2_Cl_2_ precursor, PVI-HEA copolymer, and PVI-HEA-Os(dmo-bpy)_2_Cl_2_ redox polymer. The spectra of Os(dmo-bpy)_2_Cl_2_, PVI-HEA, and PVI-HEA-Os(dmo-bpy)_2_Cl_2_ are shown in black, red, and blue, respectively. The absorption features in the longer-wavelength region are associated with the Os-containing coordination structure.

The formation of the PVI-HEA-Os(dmo-bpy)_2_Cl_2_ redox mediator was examined using UV–Vis spectroscopy. Figure 6 shows the UV–Vis absorption spectra of the Os(dmo-bpy)_2_Cl_2_ precursor, PVI-HEA copolymer, and the final PVI-HEA-Os(dmo-bpy)_2_Cl_2_ complex.

The Os(dmo-bpy)_2_Cl_2_ precursor showed characteristic absorption bands originating from the dmo-bpy ligand and the Os metal center. Strong absorption bands observed in the range of 200–300 nm are mainly related to the π–π* transition of the bipyridine-based ligand structure. In addition, a broad absorption feature extending to longer wavelengths was observed, which is associated with metal-to-ligand charge-transfer (MLCT) transitions of the Os complex.

In contrast, the PVI-HEA copolymer mainly showed absorption at 200–250 nm, while almost no clear absorption bands were observed above 300 nm. This result is consistent with the polymer structure containing imidazole and ester groups without a metal-centered charge-transfer system.

The UV–Vis spectrum of the final PVI-HEA-Os(dmo-bpy)_2_Cl_2_ complex showed absorption characteristics from the PVI-HEA copolymer and the Os precursor. In particular, the characteristic absorption bands related to the dmo-bpy ligand were maintained after complex formation. In addition, a broad absorption band was still observed in the longer-wavelength region above 300 nm, which was attributed to the Os complex.

These results suggest that the Os(dmo-bpy)_2_Cl_2_ precursor was successfully coordinated with the PVI-HEA copolymer to form the Os-based redox polymer. The retention of the Os-related absorption features after complex formation suggests that the electrochemically active Os coordination structure remained stable in the final mediator system [22,28,32,33].

**Figure 7:**
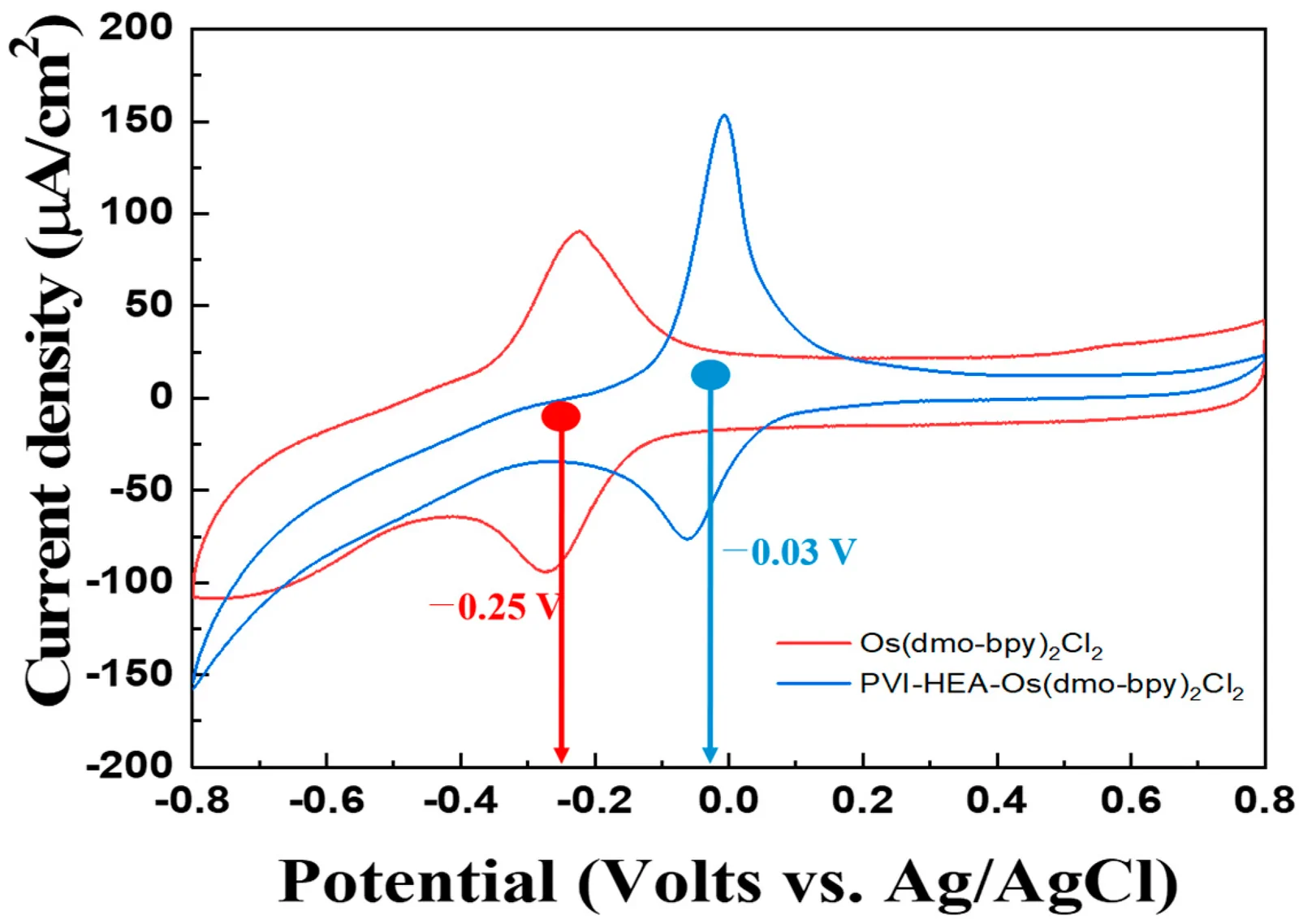
Cyclic voltammograms of the Os(dmo-bpy)_2_Cl_2_ precursor and PVI-HEA-Os(dmo-bpy)_2_Cl_2_ redox polymer recorded in 1× PBS (pH 7.4) over the potential range from −0.6 to +0.6 V versus Ag/AgCl. The voltammograms of Os(dmo-bpy)_2_Cl_2_ and PVI-HEA-Os(dmo-bpy)_2_Cl_2_ are shown in red and blue, respectively. The redox potential shifted from approximately −0.25 to −0.03 V after coordination of the Os complex with the PVI-HEA copolymer.

The formation of the PVI-HEA-Os(dmo-bpy)_2_Cl_2_ redox mediator was confirmed by CV. Figure 7 compares the CV curves of the Os(dmo-bpy)_2_Cl_2_ precursor and the PVI-HEA-Os(dmo-bpy)_2_Cl_2_ complex. The Os(dmo-bpy)_2_Cl_2_ precursor showed a redox peak near –0.25 V versus the Ag/AgCl reference electrode. The PVI-HEA-Os(dmo-bpy)_2_Cl_2_ complex exhibited a more distinct redox peak near –0.03 V, which shifted by approximately +0.22 V after coordination with the PVI-HEA copolymer.

This positive shift in redox potential is likely associated with changes in the coordination environment around the Os center after interaction with the imidazole groups of PVI-HEA. The results suggest that the chloride ligand environment of the Os(dmo-bpy)_2_Cl_2_ precursor had been partially replaced by the imidazole-containing polymer ligand, leading to the formation of the Os redox polymer structure.

In addition, the PVI-HEA-Os(dmo-bpy)_2_Cl_2_ complex showed a more defined and sharper redox response than the Os(dmo-bpy)_2_Cl_2_ precursor. Hence, the Os redox centers remained electrochemically active after incorporation into the polymer structure.

These CV results, together with the FT-IR and UV–Vis spectra, support the successful formation of the PVI-HEA-Os(dmo-bpy)_2_Cl_2_ redox mediator through coordination between the Os precursor and the PVI-HEA copolymer. The observed redox response also suggests that the synthesized Os redox polymer can function as an electrochemically active electron-transfer mediator for glucose-sensing applications [22,23,24,25,32,33].

### 3.3. Surface and Dispersion Properties of PVI-HEA-Os(dmo-bpy)_2_Cl_2_

**Figure 8:**
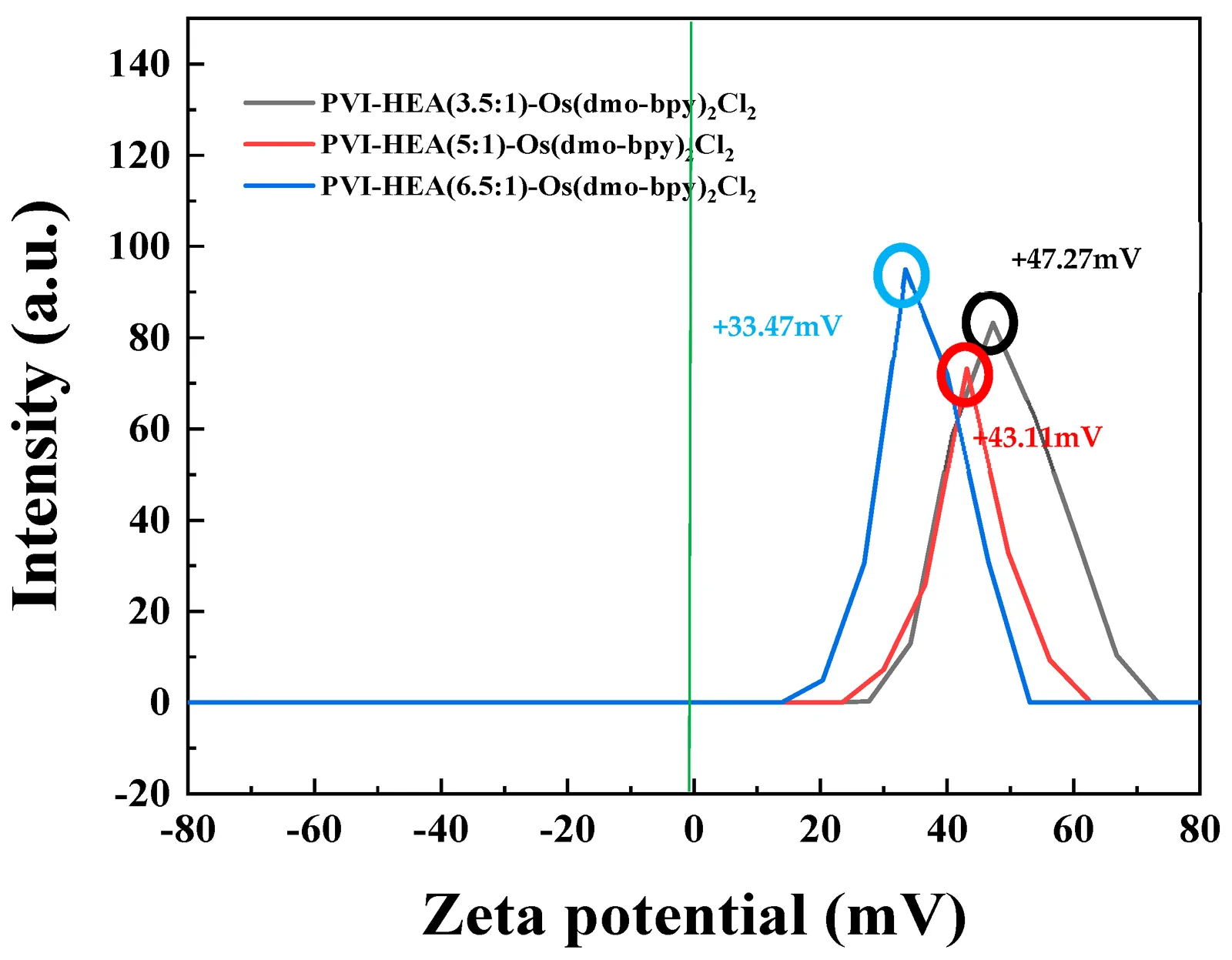
Zeta-potential distributions of PVI-HEA(3.5:1)-Os(dmo-bpy)_2_Cl_2_, PVI-HEA(5:1)-Os(dmo-bpy)_2_Cl_2_, and PVI-HEA(6.5:1)-Os(dmo-bpy)_2_Cl_2_ dispersed in deionized water and measured at approximately 25 °C. The distributions of the 3.5:1, 5:1, and 6.5:1 compositions are shown in black, red, and blue, respectively. The corresponding zeta-potential values were +47.27, +43.11, and +33.47 mV.

The dispersion properties of the synthesized PVI-HEA-Os(dmo-bpy)_2_Cl_2_ redox mediators were evaluated by zeta potential analysis. Figure 8 presents the zeta potential distributions of PVI-HEA-Os(dmo-bpy)_2_Cl_2_ complexes with different PVI-HEA compositions. The measured zeta potential values of PVI-HEA(3.5:1)-Os(dmo-bpy)_2_Cl_2_, PVI-HEA(5:1)-Os(dmo-bpy)_2_Cl_2_, and PVI-HEA(6.5:1)-Os(dmo-bpy)_2_Cl_2_ were +47.27, +43.11, and +33.47 mV, respectively. Zeta potential values higher than +30 mV or lower than −30 mV indicate relatively stable dispersion behavior because sufficient electrostatic repulsion exists between the dispersed particles or polymer complexes in aqueous solution [42].

All synthesized PVI-HEA-Os(dmo-bpy)_2_Cl_2_ complexes showed positive zeta potential values greater than +30 mV, suggesting that the Os-redox polymers formed stable cationic polymer complexes in aqueous environments. Hence, the synthesized mediators have suitable aqueous dispersion properties for electrochemical glucose sensing applications.

Differences in the zeta potential were observed depending on the PVI-HEA composition. PVI-HEA(3.5:1)-Os(dmo-bpy)_2_Cl_2_, which had the highest relative HEA content, showed the highest positive zeta potential of +47.27 mV. In contrast, PVI-HEA(5:1)-Os(dmo-bpy)_2_Cl_2_ and PVI-HEA(6.5:1)-Os(dmo-bpy)_2_Cl_2_ showed lower values of +43.11 and +33.47 mV, respectively.

Thus, the composition of the PVI-HEA copolymer influences the surface charge characteristics of the Os redox polymer. In particular, the relatively higher zeta potential observed at higher HEA content may be associated with the improved aqueous dispersion behavior and hydrated interfacial properties of the polymer complex [34,35,36,37]. These differences in dispersion behavior may also influence the electrochemical response and sensing performance of the mediator system in subsequent glucose sensing measurements [42].

**Figure 9:**
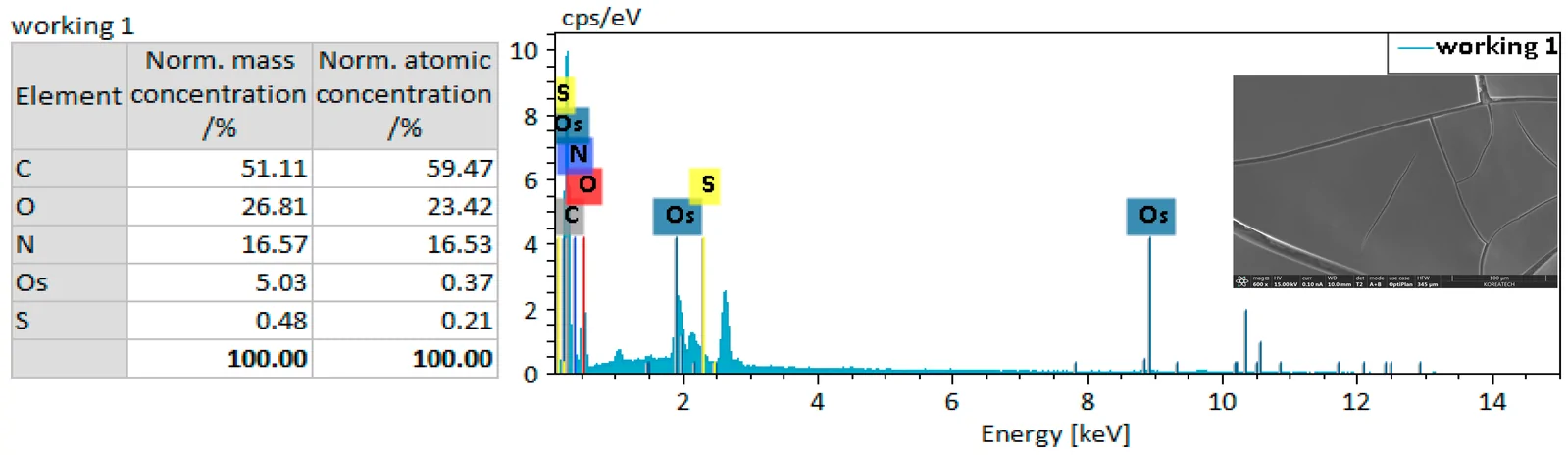
FE-SEM image and EDS spectrum of the PVI-HEA-Os(dmo-bpy)_2_Cl_2_-modified screen-printed carbon electrode. The SEM image shows the surface morphology of the mediator-coated electrode, and the EDS spectrum confirms the presence of Os within the modified electrode layer.

SEM-EDS was performed to confirm the presence of the PVI-HEA-Os(dmo-bpy)_2_Cl_2_ redox mediator on the SPCE surface. Figure 9 presents the SEM image and EDS spectrum obtained from the PVI-HEA-Os(dmo-bpy)_2_Cl_2_-modified SPCE surface.

In the SEM image, polymer-like materials were observed on the electrode surface as irregular films or aggregated structures. These surface features were attributed to the deposition of the PVI-HEA-Os(dmo-bpy)_2_Cl_2_ redox polymer during drop-casting and drying. The modified region appeared clearly different from the bare carbon surface, indicating the successful coating of the mediator layer on the SPCE.

EDS detected C, O, and Os signals from the analyzed region. Carbon can originate from the carbon substrate of the SPCE and the polymer backbone of PVI-HEA and dmo-bpy ligands. Oxygen is likely associated with the ester and hydroxyl groups of HEA as well as the methoxy groups of the dmo-bpy ligand.

Importantly, the detection of Os confirmed the presence of the Os-based redox mediator on the electrode surface. Therefore, the observed elemental composition supports the successful immobilization of the PVI-HEA-Os(dmo-bpy)_2_Cl_2_ mediator on the SPCE. This mediator layer is expected to provide electrochemically active Os redox sites for subsequent glucose sensing measurements [24,32,33].

### 3.4. Electrochemical Behavior Depending on PVI-HEA Composition

**Figure 10:**
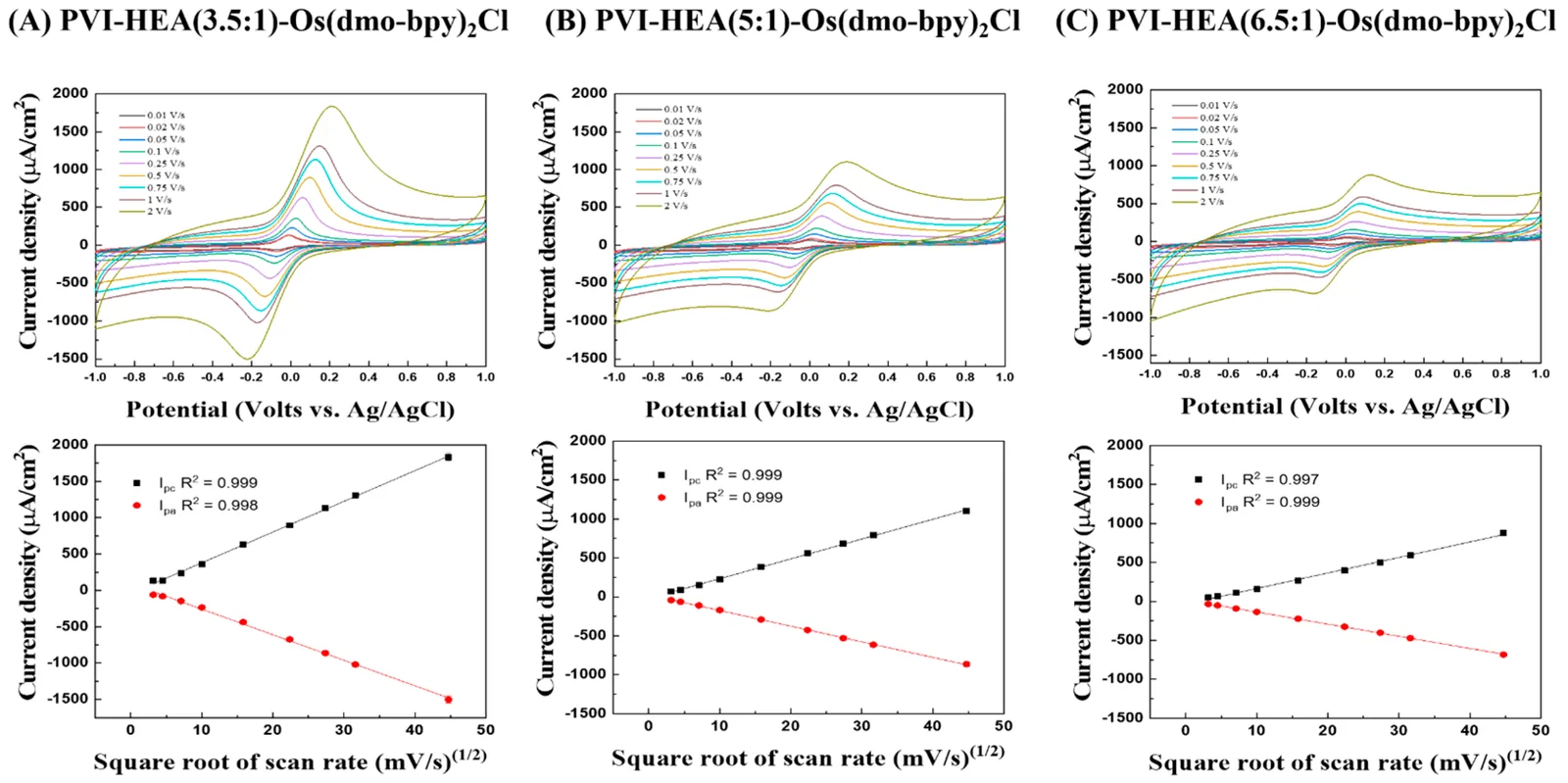
Scan-rate-dependent cyclic voltammograms and plots of the oxidation and reduction peak-current density versus the square root of the scan rate for (**A**) PVI-HEA(3.5:1)-Os(dmo-bpy)_2_Cl_2_, (**B**) PVI-HEA(5:1)-Os(dmo-bpy)_2_Cl_2_, and (**C**) PVI-HEA(6.5:1)-Os(dmo-bpy)_2_Cl_2_-modified SPCEs. The measurements were taken in 1× PBS at scan rates ranging from 0.01 to 2.0 V s^−1^. (*n* = 3).

The electrochemical behavior of PVI-HEA-Os(dmo-bpy)_2_Cl_2_ redox mediators with different PVI-HEA compositions was investigated using scan-rate-dependent CV. Figure 10 shows the CV curves and the relationship between peak current density and the square root of scan rate for PVI-HEA(3.5:1)-Os(dmo-bpy)_2_Cl_2_, PVI-HEA(5:1)-Os(dmo-bpy)_2_Cl_2_, and PVI-HEA(6.5:1)-Os(dmo-bpy)_2_Cl_2_ electrodes.

For all mediator compositions, the oxidation and reduction peak currents increased as the scan rate increased. Hence, the Os redox centers in the synthesized PVI-HEA-Os(dmo-bpy)_2_Cl_2_ complexes remained electrochemically active and repeatedly participated in redox reactions during the electrochemical measurements. The gradual increase in peak current as the scan rate increased also suggests that the synthesized Os redox polymers can function as active electron-transfer mediators under glucose-sensing conditions.

In addition, the oxidation and reduction peak currents showed an approximately linear relationship with the square root of the scan rate. The calculated linear fits showed high correlation coefficients (R^2^ = 0.997–0.999) for all compositions. This behavior suggests that the electrochemical response of the mediator system is strongly associated with diffusion-limited charge-transfer behavior within the polymer layer [39,40,41].

The corresponding oxidation and reduction slopes and intercepts for electrodes (A), (B), and (C), obtained from the linear regression analysis, are summarized in Table 1. Among the three compositions, the PVI-HEA(3.5:1)-Os(dmo-bpy)_2_Cl_2_ electrode showed the highest redox peak current density, followed by PVI-HEA(5:1)-Os(dmo-bpy)_2_Cl_2_ and PVI-HEA(6.5:1)-Os(dmo-bpy)_2_Cl_2_. The decrease in peak current at a lower HEA content suggests that the PVI-HEA composition influences the electrochemical behavior of the Os redox polymer.

**Table 1:** Linear regression parameters obtained from the scan rate dependent cyclic voltammetric analysis of PVI-HEA-Os(dmo-bpy)_2_Cl_2_-modified SPCEs.

	(A)	(B)	(C)
Oxidation Slope	−35.142 ± 0.6216	−20.083 ± 0.1450	−15.570 ± 0.0977
Intercept	93.249 ± 14.174	25.576 ± 3.3069	18.312 ± 2.2291
Reduction Slope	42.222 ± 0.5879	25.437 ± 0.2778	19.896 ± 0.3938
Intercept	−41.804 ± 13.406	−21.263 ± 6.3350	−33.559 ± 8.9802

The relatively higher redox response observed for PVI-HEA(3.5:1)-Os(dmo-bpy)_2_Cl_2_ may be related to the higher HEA content in the polymer structure. The hydroxyethyl groups of HEA can provide a more hydrophilic polymer environment [34,35,36], which may help improve the accessibility of redox-active sites and favorable interfacial charge-transfer behavior under aqueous electrolyte conditions [39,40,41]. In contrast, a lower HEA content may reduce the hydrated characteristics of the polymer layer, resulting in a relatively weaker electrochemical response [34,35,36].

These results suggest that the hydrophilic composition of the PVI-HEA copolymer influences the electrochemical characteristics of the Os redox mediator system and may affect the subsequent glucose sensing performance.

**Figure 11:**
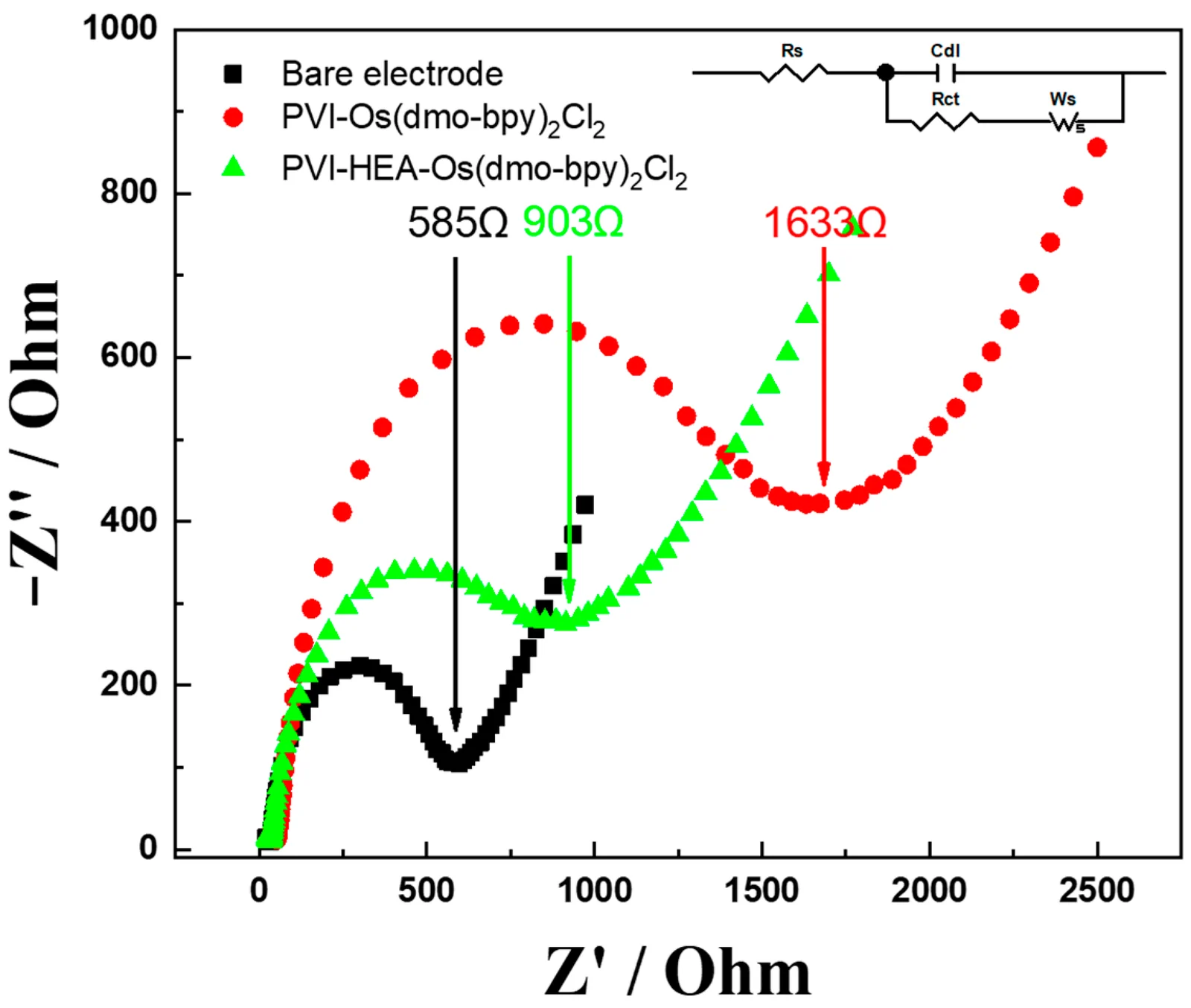
Nyquist plots of the bare SPCE, PVI-Os(dmo-bpy)_2_Cl_2_-modified SPCE, and PVI-HEA-Os(dmo-bpy)_2_Cl_2_-modified SPCE recorded in 0.5 M KCl containing 2.0 mM [Fe(CN)_6_]^3−^/^4−^. EIS measurements were taken at 0.266 V versus Ag/AgCl with an AC amplitude of 5 mV over a frequency range of 1 Hz–100 kHz.

EIS was performed to examine the effects of the PVI-HEA-Os(dmo-bpy)_2_Cl_2_ redox mediator on the interfacial charge-transfer behavior of the electrode surface. Figure 11 shows the Nyquist plots of the bare electrode, PVI-Os(dmo-bpy)_2_Cl_2_-modified electrode, and PVI-HEA-Os(dmo-bpy)_2_Cl_2_-modified electrode measured in KCl electrolyte containing the [Fe(CN)_6_]^3-/4-^ redox probe.

In the Nyquist plots, the diameter of the semicircle is related to charge-transfer resistance (Rct) at the electrode/electrolyte interface. The bare electrode showed the lowest Rct value of approximately 585 Ω, indicating relatively rapid charge transfer at the unmodified electrode surface [40].

After modification with Os redox polymers, the semicircle diameter increased, indicating changes in interfacial charge-transfer behavior caused by the polymer layers on the electrode surface. The PVI-Os(dmo-bpy)_2_Cl_2_-modified electrode showed the highest Rct value of approximately 1633 Ω, while the PVI-HEA-Os(dmo-bpy)_2_Cl_2_-modified electrode showed a lower Rct value of approximately 903 Ω.

The lower charge-transfer resistance observed for the PVI-HEA-Os(dmo-bpy)_2_Cl_2_-modified electrode compared with the PVI-Os(dmo-bpy)_2_Cl_2_-modified electrode suggests that the introduction of HEA influenced the interfacial electrochemical properties of the polymer layer. This behavior may be associated with the hydrophilicity of the HEA-containing polymer structure [34,35,36,37]. The hydroxyethyl groups of HEA can provide a hydrated polymer environment that may facilitate electrolyte penetration and improve the accessibility of redox probes to the electrode interface [40].

As a result, the PVI-HEA-containing Os redox polymer showed relatively improved interfacial charge-transfer behavior compared to the PVI-only Os redox polymer system. These results are consistent with the scan-rate-dependent CV analysis and suggest that the hydrophilic polymer composition can influence the electrochemical behavior of the mediator layer under aqueous conditions.

Although the polymer-modified electrodes showed higher Rct values than the bare electrode because of the additional polymer coating layer [40], the HEA-containing mediator system exhibited lower resistance than the PVI-only mediator system, suggesting that the HEA structure may provide more favorable electrochemical interface characteristics for glucose sensing applications [22,28,32,33].

### 3.5. Performance of PVI-HEA-Os(dmo-bpy)_2_Cl_2_/GDH/PEGDGE/SPCE

**Figure 12:**
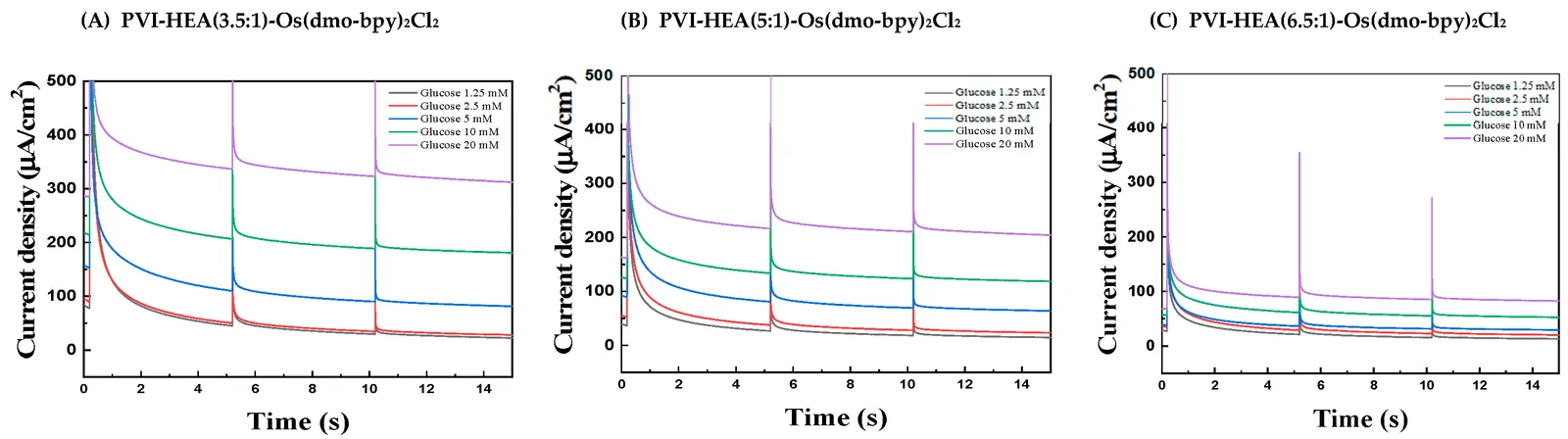
Multi-potential-step responses of MET/FAD-GDH/PEGDGE electrodes containing (**A**) PVI-HEA(3.5:1)-Os(dmo-bpy)_2_Cl_2_, (**B**) PVI-HEA(5:1)-Os(dmo-bpy)_2_Cl_2_, and (**C**) PVI-HEA(6.5:1)-Os(dmo-bpy)_2_Cl_2_ at glucose concentrations of 1.25, 2.5, 5, 10, and 20 mM in 1× PBS (pH 7.4). Potentials of 0.1, 0.2, and 0.3 V versus Ag/AgCl were applied sequentially for 5 s each.

The glucose-sensing performance of PVI-HEA-Os(dmo-bpy)_2_Cl_2_ redox mediators with different PVI-HEA compositions was evaluated using SPCE-based enzyme electrodes containing FAD-dependent glucose dehydrogenase (FAD-GDH) and PEGDGE. Figure 12 shows the multi-potential step (MPS) responses of PVI-HEA(3.5:1)-Os(dmo-bpy)_2_Cl_2_, PVI-HEA(5:1)-Os(dmo-bpy)_2_Cl_2_, and PVI-HEA(6.5:1)-Os(dmo-bpy)_2_Cl_2_-based electrodes at different glucose concentrations.

Glucose concentrations of 1.25, 2.5, 5, 10, and 20 mM were tested. During the MPS measurements, potentials of 0.1 V, 0.2 V, and 0.3 V versus Ag/AgCl were applied sequentially at 0–5 s, 5–10 s, and 10–15 s, respectively.

For all electrode compositions, the current density increased gradually as the glucose concentration increased. This result suggests that the synthesized PVI-HEA-Os(dmo-bpy)_2_Cl_2_ mediators successfully transferred electrons generated from the enzymatic oxidation of glucose [8,9] to the electrode surface [14,15,16,17,18,19,20,22,23,24,25].

Differences in the glucose response were observed depending on the PVI-HEA composition. Among the electrodes tested, the PVI-HEA(3.5:1)-Os(dmo-bpy)_2_Cl_2_-based electrode showed the strongest current response over the entire glucose concentration range. In contrast, the PVI-HEA(6.5:1)-Os(dmo-bpy)_2_Cl_2_-based electrode showed the lowest current response. The PVI-HEA(5:1)-Os(dmo-bpy)_2_Cl_2_ electrode showed intermediate behavior between the two compositions.

The stronger glucose response observed for the PVI-HEA(3.5:1)-Os(dmo-bpy)_2_Cl_2_ electrode is consistent with the CV and EIS results [39,40,41,42]. A relatively higher HEA content may provide a more favorable hydrated-polymer environment that improves interfacial charge transfer and accessibility of Os redox sites during enzymatic glucose oxidation [34,35,36,37].

In addition, all electrodes showed stable stepwise current responses to repeated potential changes, suggesting that the Os redox polymer layers maintained electrochemical activity under glucose-sensing conditions. Hence, the composition of the PVI-HEA copolymer influences the glucose-sensing behavior of the Os redox mediator system, and a higher HEA content may help improve the electrochemical glucose-sensing performance.

**Figure 13:**
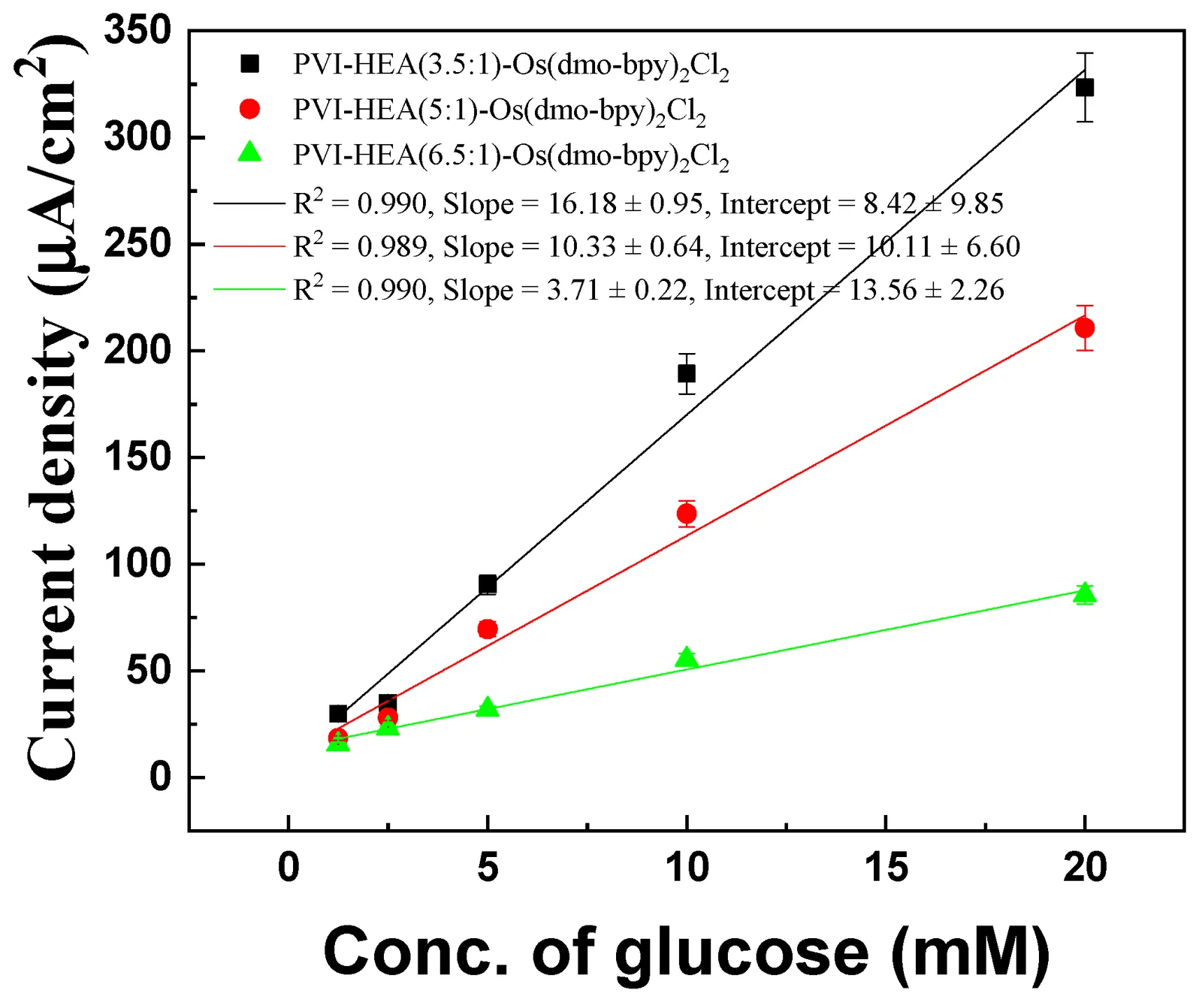
Calibration plots of glucose current density measured at 0.2 V versus Ag/AgCl using FAD-GDH/PEGDGE electrodes containing PVI-HEA(3.5:1)-Os(dmo-bpy)_2_Cl_2_, PVI-HEA(5:1)-Os(dmo-bpy)_2_Cl_2_, or PVI-HEA(6.5:1)-Os(dmo-bpy)_2_Cl_2_. The glucose concentration range was 1.25–20 mM in 1× PBS (pH 7.4). The corresponding linear-regression equations and coefficients of determination are shown in the plots. (*n* = 3).

Figure 13 presents the glucose calibration curves obtained from the current densities measured at 0.2 V during the multi-potential-step (MPS) measurements. All PVI-HEA-Os(dmo-bpy)_2_Cl_2_-based electrodes showed linear increases in current density over the glucose concentration range of 1.25–20 mM, suggesting that the Os redox polymer systems can provide concentration-dependent electrochemical glucose responses.

Among the tested electrodes, the PVI-HEA(3.5:1)-Os(dmo-bpy)_2_Cl_2_ electrode showed the highest sensitivity, with a linear fitting coefficient of R^2^ = 0.990 and a sensitivity of 16.18 μA cm−2 mM^−1^. The PVI-HEA(5:1)-Os(dmo-bpy)_2_Cl_2_ electrode showed intermediate sensing performance with R^2^ = 0.989 and a sensitivity of 10.33 μA cm^−2^ mM^−1^. In contrast, the PVI-HEA(6.5:1)-Os(dmo-bpy)_2_Cl_2_ electrode showed the lowest sensitivity, with R^2^ = 0.990 and a sensitivity of 3.71 μA cm−2 mM^−1^.

The differences in glucose sensitivity depending on the PVI-HEA composition suggest that the hydrophilic polymer composition influences the electrochemical glucose-sensing behavior of the Os redox mediator system. In particular, the higher sensitivity observed for the PVI-HEA(3.5:1)-Os(dmo-bpy)_2_Cl_2_ electrode is consistent with the higher redox peak current and lower interfacial charge-transfer resistance observed in CV and EIS.

The relatively higher HEA content in the PVI-HEA(3.5:1) structure may provide a more favorable hydrated-polymer environment around the Os redox sites and enzyme layer. This hydrated environment may help improve the interfacial charge-transfer behavior and enable more efficient electron transfer during enzymatic glucose oxidation. On the other hand, decreasing the HEA content led to reduced glucose sensitivity, suggesting that the polymer composition affects the electrochemical accessibility and sensing behavior of the mediator layer [34,35,36,37].

Importantly, all electrodes maintained good linearity over the tested glucose concentration range, suggesting that the synthesized Os redox polymer systems can function as stable electron-transfer mediators for glucose sensing applications. These results show that controlling the HEA composition of the PVI-HEA copolymer is an effective approach for tuning the electrochemical glucose sensing performance of Os-based redox polymers.

**Figure 14:**
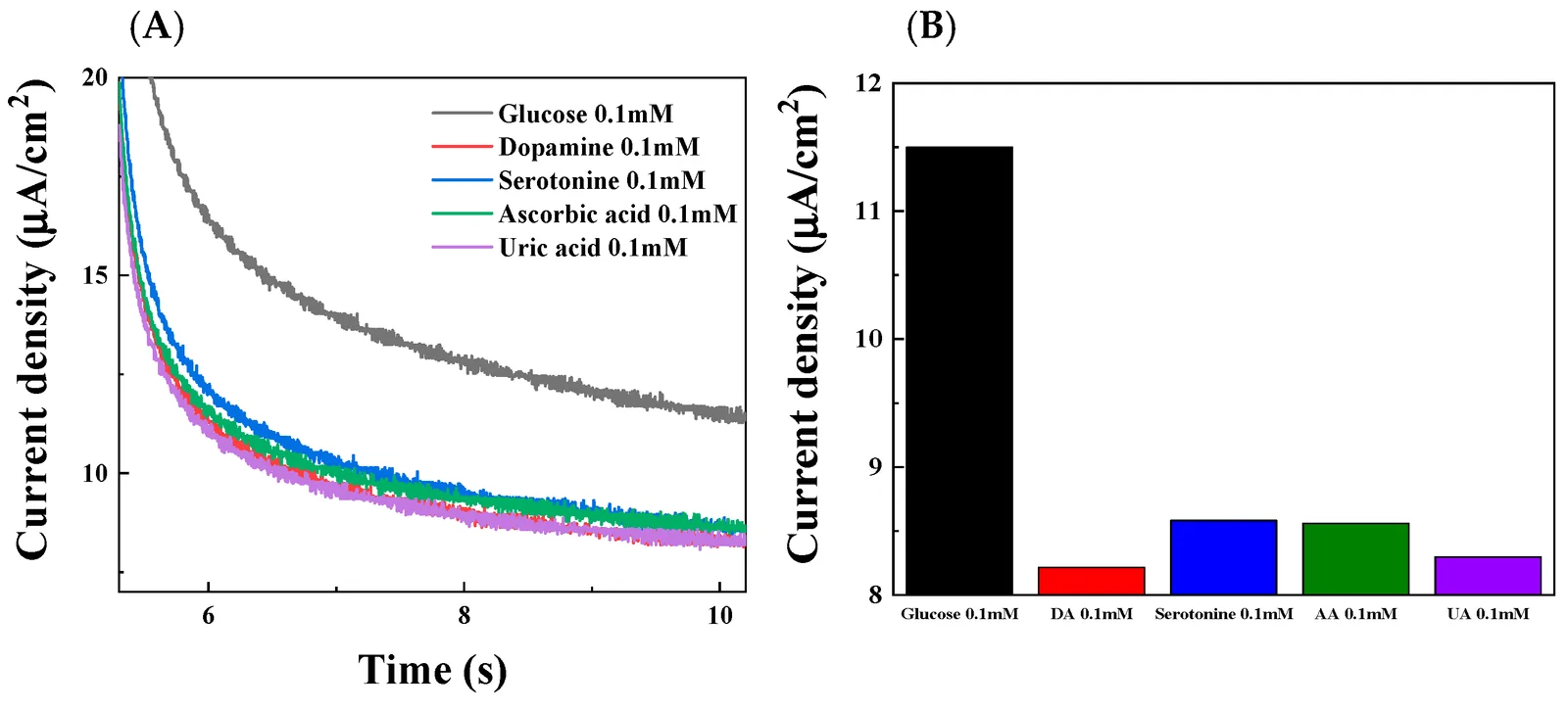
Selectivity of the FAD-GDH/PVI-HEA(3.5:1)-Os(dmo-bpy)_2_Cl_2_/PEGDGE-modified SPCE toward glucose and representative electroactive interferents. (**A**) Chronoamperometric current–time responses obtained at 0.2 V versus Ag/AgCl for 5 s for 0.1 mM glucose and 0.1 mM ascorbic acid (AA), uric acid (UA), dopamine (DA), and serotonin (5-HT) in 1× PBS (pH 7.4). (**B**) Comparison of the current densities measured at 0.2 V versus Ag/AgCl. Glucose, AA, UA, DA, and 5-HT are represented in black, green, violet, red, and blue, respectively.

The selectivity of the PVI-HEA-Os(dmo-bpy)_2_Cl_2_/GDH/PEGDGE/SPCE electrode was evaluated by comparing the current responses toward glucose and representative electrochemical interferents commonly found in biological fluids, including AA, UA, DA, and 5-HT. Figure 14 presents the amperometry responses obtained using the optimized PVI-HEA(3.5:1)-Os(dmo-bpy)_2_Cl_2_-based electrode [5,8,9,10,21,44].

Under the same measurement conditions, glucose produced a significantly higher current response than the tested interferents. In contrast, AA, UA, DA, and 5-HT showed relatively low current responses at 0.2 V. The bar graph in Figure 14B also clearly shows that the glucose response was higher than that of the interfering species.

The low interference response is likely related to the relatively low operating potential of the Os redox mediator system and the substrate selectivity of the FAD-GDH enzyme [8,9,10,12,13]. FAD-GDH selectively catalyzes glucose oxidation, while the PVI-HEA-Os(dmo-bpy)_2_Cl_2_ mediator transfers electrons from the enzyme to the electrode at a relatively low potential. As a result, the oxidation of electroactive interferents, which generally occurs at higher potentials, can be reduced under the present sensing conditions [5,8,9,10,21,44].

These results suggest that the PVI-HEA(3.5:1)-Os(dmo-bpy)_2_Cl_2_/GDH/PEGDGE/SPCEs maintained selective electrochemical responses toward glucose even in the presence of common biological interferents [5,8,9,10,21,44]. The observed interference resistance suggests that the synthesized Os redox polymer system may be suitable for electrochemical glucose sensing under biologically relevant conditions and may have potential CGM-related applications [2,3].

**Figure 15:**
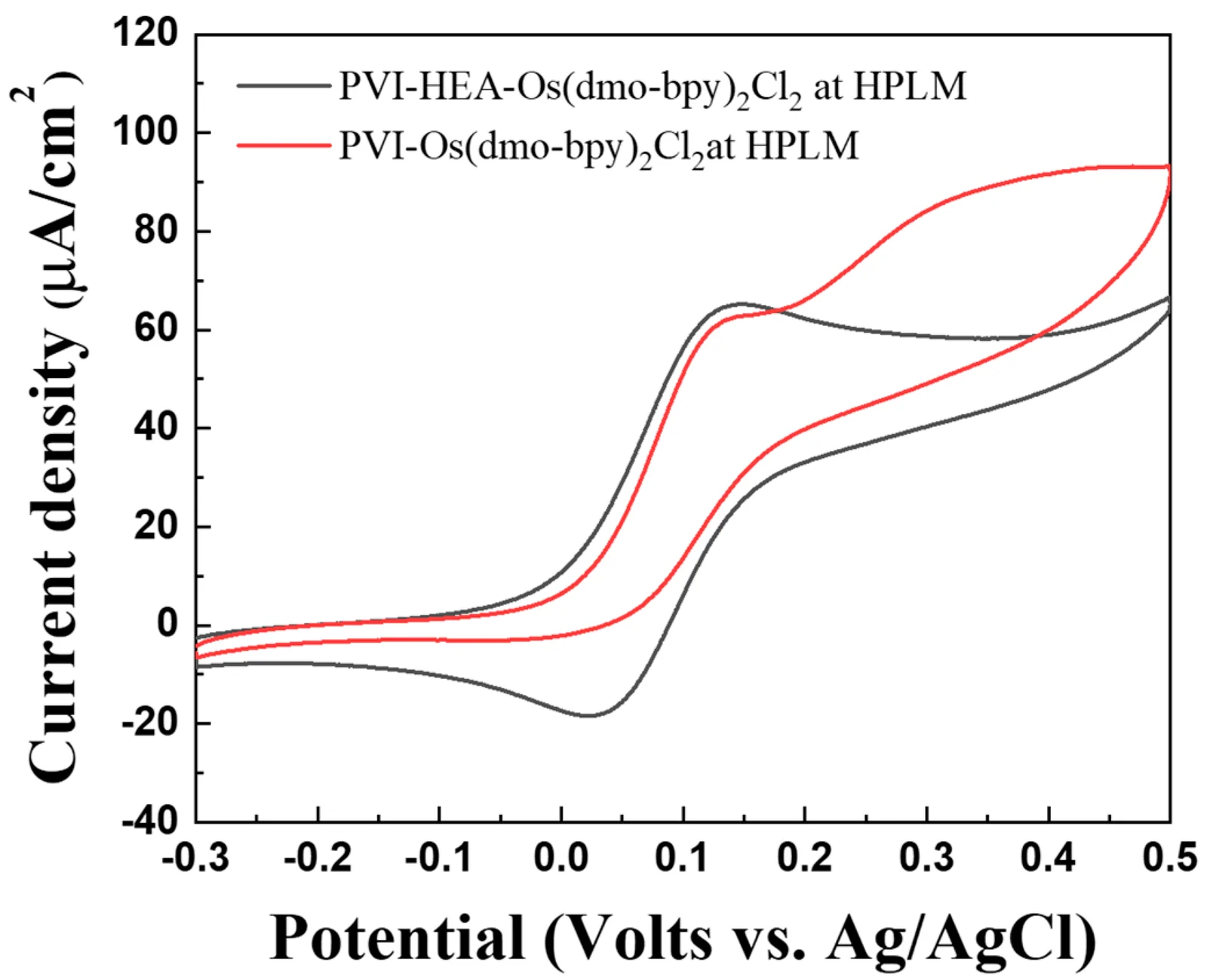
Cyclic voltammograms of PVI-HEA(3.5:1)-Os(dmo-bpy)_2_Cl_2_/FAD-GDH/PEGDGE/SPCE and PVI-Os(dmo-bpy)_2_Cl_2_/FAD-GDH/PEGDGE/SPCE measured in HPLM containing 5.5 mM glucose at a scan rate of 0.02 V s^−1^. The PVI-HEA-based and conventional PVI-based Os redox-polymer electrodes are shown in black and red, respectively.

The applicability of the synthesized mediator system under biologically relevant conditions was evaluated by comparing the electrochemical behavior of PVI-HEA(3.5:1)-Os(dmo-bpy)_2_Cl_2_/GDH/PEGDGE/SPCE and conventional PVI-Os(dmo-bpy)_2_Cl_2_/GDH/PEGDGE/SPCE in HPLM. Figure 15 shows the cyclic voltammograms of the two mediator-based enzyme electrodes measured under the same HPLM conditions [45].

The PVI-HEA(3.5:1)-Os(dmo-bpy)_2_Cl_2_-based electrode exhibited a more stable and controlled electrochemical response in HPLM [45]. After the oxidation current increased in the low-potential region, the current response became relatively steady over the higher-potential region. Hence, the PVI-HEA-Os-based mediator maintained its redox activity while suppressing an excessive background current in the complex plasma-like matrix [32,33,45].

In contrast, the conventional PVI-Os(dmo-bpy)_2_Cl_2_-based electrode showed a continuous increase in current density at higher potentials. This additional current increase is likely associated with nonspecific oxidation processes or matrix-derived background currents from components present in HPLM. The glucose-dependent response of the conventional PVI-Os system may be more strongly affected by matrix interference because this high-potential current response is not clearly separated from the mediator-based catalytic signal [21,44,45].

The improved behavior of the PVI-HEA-Os(dmo-bpy)_2_Cl_2_ system can be attributed to the hydrophilic polymer environment introduced by HEA units [34,35,36,37]. The hydroxyethyl groups in PVI-HEA can form a hydrated mediator layer around the enzyme and redox polymer, which helps stabilize the enzyme–mediator–electrode interface under plasma-like conditions [45]. This hydrated polymer environment may also reduce nonspecific interactions between the electrode surface and matrix components, resulting in a lower background current and a more reliable electrochemical response [34,35,36,37,45].

These results show that the HEA-containing osmium redox polymer provides improved matrix tolerance compared to the conventional PVI-based osmium mediator. Therefore, PVI-HEA(3.5:1)-Os(dmo-bpy)_2_Cl_2_ is a promising mediator structure for enzymatic glucose sensing under biologically relevant conditions.The aqueous storage stability of the optimized PVI-HEA(3.5:1)-Os(dmo-bpy)_2_Cl_2_/FAD-GDH/PEGDGE/SPCE was evaluated over six days under repeated daily measurement conditions. Three independently prepared electrodes were immersed in 1× PBS for storage between measurements, and the current response measured on day 0 was normalized to 100%.

**Figure 16:**
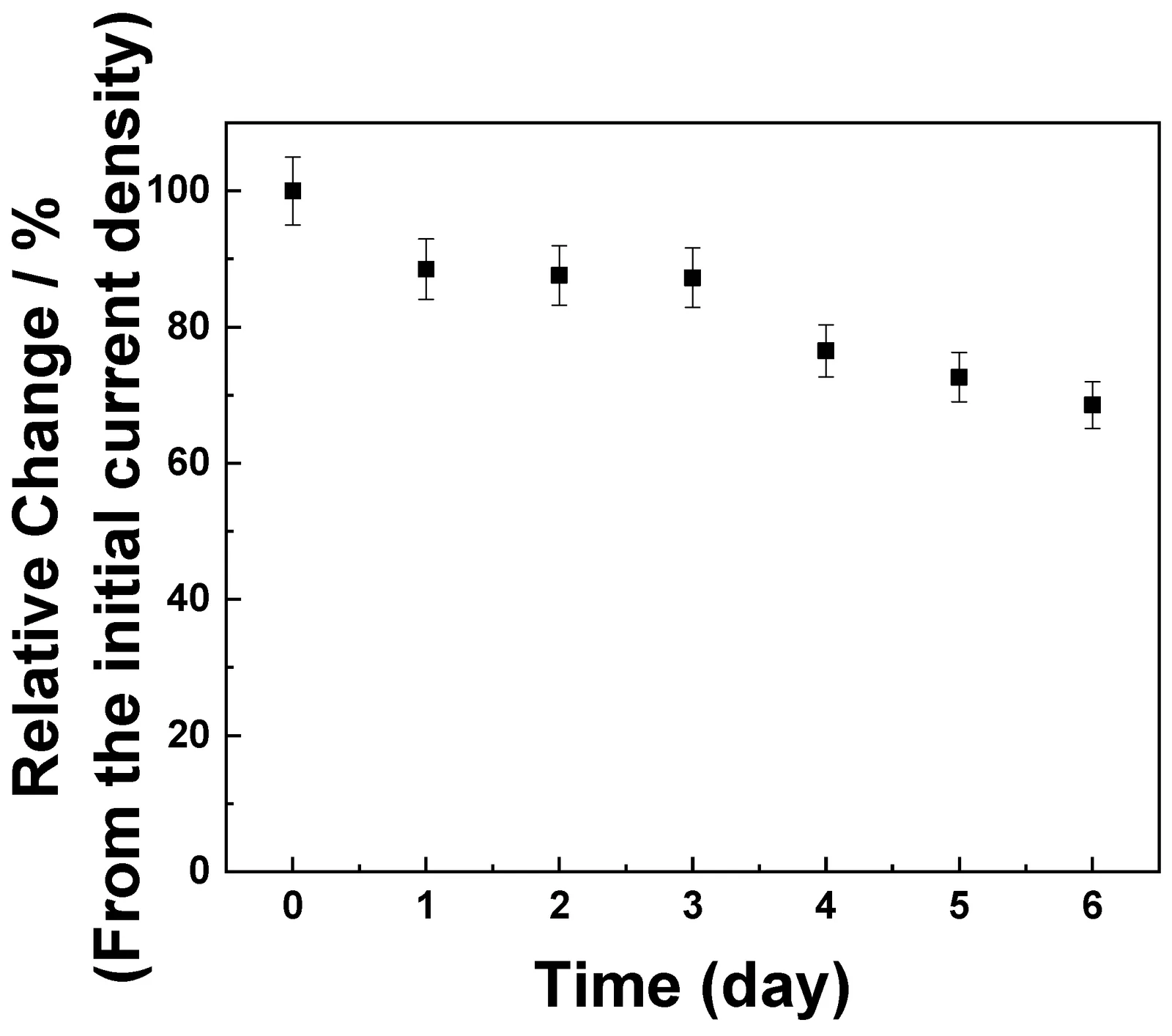
Six-day aqueous storage stability of the PVI-HEA(3.5:1)-Os(dmo-bpy)_2_Cl_2_/FAD-GDH/PEGDGE-modified SPCE under repeated daily measurement conditions. Three independently prepared electrodes were stored immersed in 1× PBS between measurements, and their current responses were measured once daily using 5.5 mM glucose at 0.2 V versus Ag/AgCl. The current response measured on day 0 was normalized to 100%. The data are presented as the mean ± standard deviation (*n* = 3).

Approximately 85% of the initial current response was retained during the first three days, as shown in Figure 16. Thereafter, the relative response gradually decreased to approximately 76.5%, 72.6%, and 68.6% on days 4, 5, and 6, respectively. Despite the progressive decrease observed after day 3, the electrodes maintained measurable glucose-sensing activity throughout the six-day test period.

The same electrodes were stored in 1× PBS and measured once daily. Hence, these results represent aqueous storage stability under repeated measurement conditions rather than uninterrupted continuous operational stability.

**Figure 17:**
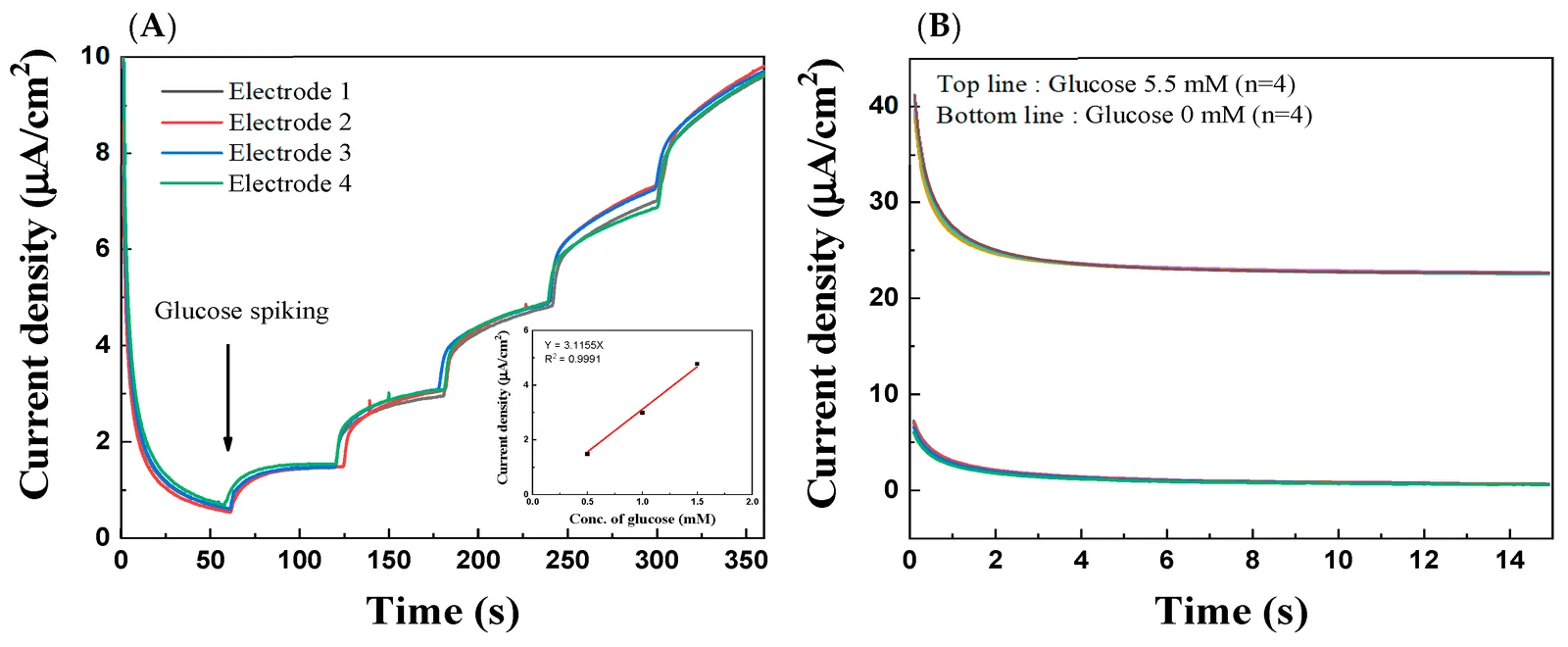
Electrode-to-electrode reproducibility and repeatability of the PVI-HEA(3.5:1)-Os(dmo-bpy)_2_Cl_2_/FAD-GDH/PEGDGE glucose sensor. (**A**) Amperometric current–time (i–t) responses obtained using four independently prepared electrodes during successive glucose additions. (**B**) Repeatability evaluated by four consecutive measurements using the same electrode in 1× PBS containing 5.5 mM glucose (top line) and in glucose-free 1× PBS (bottom line). The inset shows the low-concentration calibration curve constructed from the steady-state current densities measured at 0.5–1.5 mM glucose. The linear regression equation was y = 3.1155x, with R^2^ = 0.9991. The limit of detection (LOD) and limit of quantification (LOQ) were calculated according to the ICH Q2(R2) guideline.

The electrode-to-electrode reproducibility of the PVI-HEA(3.5:1)-Os(dmo-bpy)_2_Cl_2_/FAD-GDH/PEGDGE sensor was evaluated using four independently fabricated electrodes (Figure 17A). Amperometric measurements were initiated in 5.0 mL of 1× PBS, followed by successive additions of 2 μL of a 1.25 M glucose stock solution. Each addition nominally increased the glucose concentration by 0.5 mM, producing a concentration range of 0–2.5 mM. The four independently prepared electrodes exhibited comparable stepwise increases in current density, indicating reproducible sensor fabrication and glucose-response characteristics. The inset of Figure 17A presents the low-concentration calibration curve constructed using the steady-state current densities obtained at 0.5, 1.0, and 1.5 mM glucose. The calibration equation was y = 3.1155x, with R^2^ = 0.9991, demonstrating good linearity within the low-concentration range. According to the ICH Q2(R2) guideline, the LOD and LOQ were calculated using 3.3σ/S and 10σ/S, respectively, where σ is the standard deviation of the blank response and S is the slope of the low-concentration calibration curve. The resulting LOD and LOQ were 0.0654 and 0.1984 mM, respectively (Table 2). Repeatability was evaluated by performing four consecutive measurements using the same electrode in glucose-free 1× PBS and in 1× PBS containing 5.5 mM glucose (Figure 17B). The mean current density was 0.6596 ± 0.04625 μA cm^−2^ at 0 mM glucose and 22.6218 ± 0.07083 μA cm^−2^ at 5.5 mM glucose (mean ± standard deviation, *n* = 4). The corresponding relative standard deviations were 7.01% and 0.31%, respectively (Table 3). Although the blank response exhibited a relatively high %RSD because of its low mean current density, its absolute standard deviation remained small. The low %RSD obtained at 5.5 mM glucose indicates excellent repeatability of the glucose-dependent sensor response.

**Table 2:** LOD and LOQ values using the standard deviation of the background signal according to the ICH Q2(R2) guidelines. (*n* = 4).

Test Number	BKG Signal	ICH Q2(R2) Guideline	0.5–1.5 mM Calibration Curve
1	0.69745	LOD = 3.3 s/m	Y = 3.1155X
2	0.62672	LOQ = 10 s/m	R^2^ = 0.9991
3	0.70216	Standard deviation(s) = 0.06181 μA/cm^2^	LOD = 0.0654 mM
4	0.77800	Sensitivity(m) = 3.1155 μA/cm^2^ × mM	LOQ = 0.1984 mM

**Table 3:** Repeatability of the optimized PVI-HEA(3.5:1)-Os(dmo-bpy)_2_Cl_2_/FAD-GDH/PEGDGE glucose sensor. (*n* = 4).

Test Number	0 mM	5.5 mM	%RSD
1	0.6963	22.7073	Standard deviation (0 mM) = 0.04625 μA/cm^2^
2	0.7028	22.6012	Standard deviation (5.5 mM) = 0.07083 μA/cm^2^
3	0.6204	22.5383	%RSD = 7.01% (0 mM)
4	0.6188	22.6 404	%RSD = 0.31% (5.5 mM)

**Figure 18:**
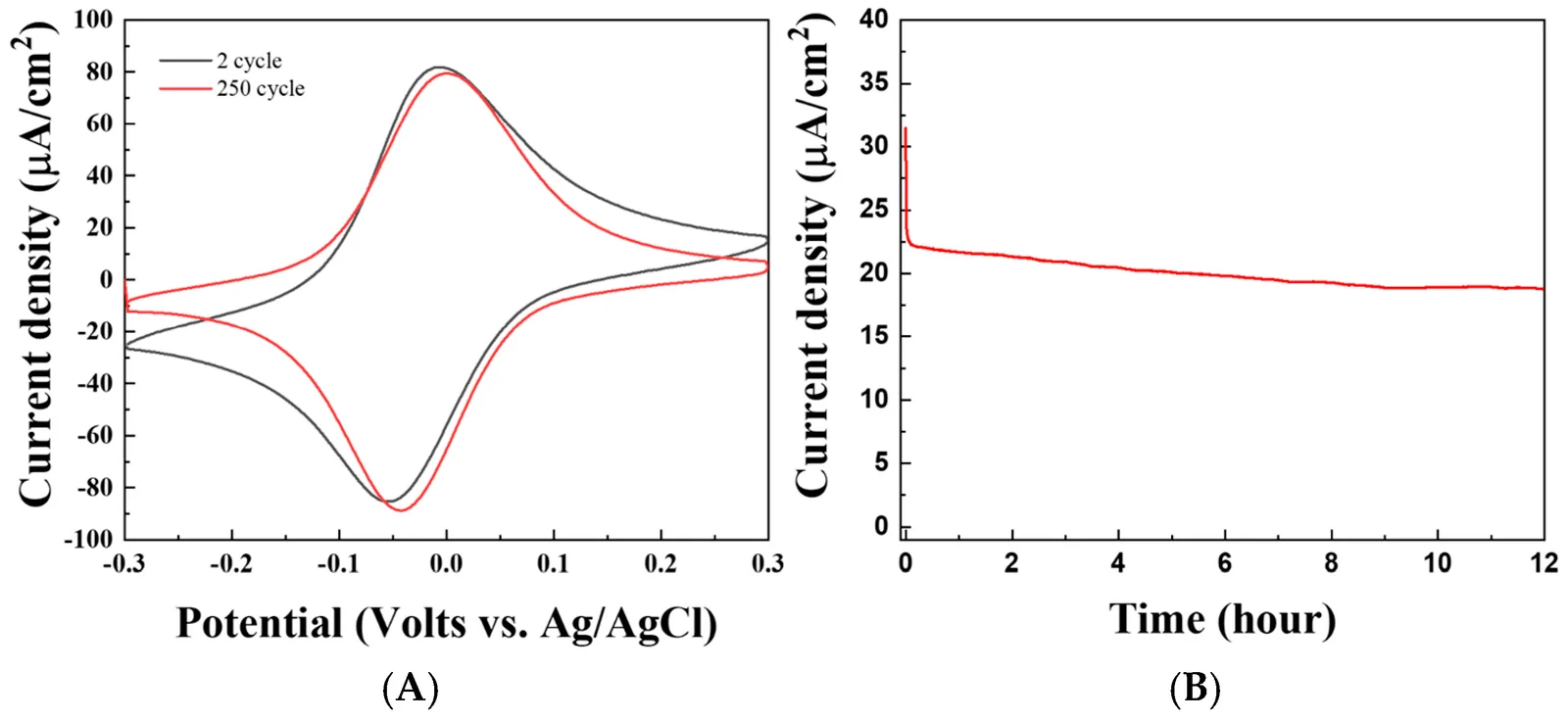
Operational stability of the PVI-HEA(3.5:1)-Os(dmo-bpy)_2_Cl_2_/FAD-GDH/PEGDGE sensor. (**A**) Comparison of cyclic voltammograms recorded at the 1st and 250th cycles, demonstrating the electrochemical stability of the immobilized mediator layer during repeated potential cycling. (**B**) Continuous amperometric response recorded for 12 h in 1× PBS containing 5.5 mM glucose under static batch conditions.

The operational stability of the PVI-HEA(3.5:1)-Os(dmo-bpy)_2_Cl_2_/FAD-GDH/PEGDGE glucose sensor. was evaluated by repeated potential cycling and continuous amperometric measurement (Figure 18). As shown in Figure 18A, the cyclic voltammograms recorded at the 1st and 250th cycles exhibited similar redox profiles and peak currents, indicating that the electrochemical activity of the immobilized osmium mediator was largely maintained during repeated cycling. Continuous amperometric measurement was subsequently performed for 12 h in 1× PBS containing 5.5 mM glucose (Figure 18B). The current density gradually decreased from approximately 22.3 to 18.8 μA cm^−2^, corresponding to an apparent signal retention of 84.3% and a signal decrease of 15.7%. However, this decrease should not be attributed solely to deterioration of the sensor layer. Because the experiment was conducted in a static batch solution without glucose replenishment, glucose was continuously consumed through the FAD-GDH-catalyzed oxidation reaction. Consequently, progressive depletion of glucose, particularly within the diffusion layer near the electrode surface and potentially in the bulk solution, likely contributed to the observed decrease in current. Other factors, including hydration and equilibration of the polymer–enzyme layer, changes in mass transport, partial enzyme deactivation, or loss of immobilized components, may also have affected the response.

## 4. Discussion and Conclusions

PVI-HEA-Os(dmo-bpy)_2_Cl_2_ redox polymers were designed as electron-transfer mediators for FAD-GDH-based glucose sensors, and the influence of the PVI-HEA copolymer composition on the electrochemical and glucose-sensing performance was systematically investigated. FT-IR, ^1^H-NMR, and UV–Vis spectroscopy supported the successful synthesis of the PVI-HEA copolymers and the incorporation of the Os complex into the imidazole-containing polymer structure. In addition, the apparent redox potential shifted from approximately −0.25 V for the Os(dmo-bpy)_2_Cl_2_ precursor to approximately −0.03 V after formation of the PVI-HEA-Os(dmo-bpy)_2_Cl_2_ complex. This shift, together with the spectroscopic results, is consistent with a change in the coordination environment of the Os center following its interaction with the PVI-HEA copolymer [22,23,24,25,32,33].

The electrochemical behavior of the resulting Os redox polymers depended strongly on the PVI-to-HEA composition. All three mediator systems exhibited positive zeta potentials and stable dispersion behavior in aqueous media, while scan-rate-dependent CV confirmed that the polymer-bound Os centers remained electrochemically active. Among the compositions examined, PVI-HEA(3.5:1)-Os(dmo-bpy)_2_Cl_2_, which contained the highest relative HEA content, produced the highest redox peak-current density. It also exhibited a lower charge-transfer resistance than the conventional PVI-Os-modified electrode under the ferri/ferrocyanide-probe EIS conditions. These results are consistent with the interpretation that the HEA-containing copolymer composition influences the aqueous accessibility and interfacial electrochemical behavior of the polymer-bound redox sites. However, because water uptake and film hydration were not measured directly, the contribution of HEA should be interpreted from the observed composition-dependent electrochemical behavior rather than as direct evidence of a specific hydration mechanism [34,35,36,37,38,39,40,41,42].

The glucose-sensing performance followed the same composition-dependent trend. All PVI-HEA-Os/FAD-GDH/PEGDGE electrodes exhibited linear glucose-dependent responses over 1.25–20 mM. The PVI-HEA(3.5:1)-Os(dmo-bpy)_2_Cl_2_-based electrode showed the highest sensitivity of 16.18 μA cm^−2^ mM^−1^, whereas the corresponding sensitivities of the PVI-HEA(5:1)-and PVI-HEA(6.5:1)-based electrodes were 10.33 and 3.71 μA cm^−2^ mM^−1^, respectively. Because the Os precursor, enzyme, crosslinker, and electrode-preparation procedure were maintained under the same conditions, these differences support the conclusion that the copolymer composition itself is an important design parameter governing the apparent electron-transfer and catalytic glucose-sensing behavior of the enzyme–mediator layer.

Additional analytical validation of the optimized PVI-HEA(3.5:1)-Os(dmo-bpy)_2_Cl_2_/FAD-GDH/PEGDGE sensor was performed using four independently prepared electrodes. The electrode-to-electrode current–time profiles showed consistent stepwise responses during successive glucose additions, supporting the reproducibility of the electrode-fabrication and measurement procedures. The low-concentration calibration curve obtained over 0.5–1.5 mM was described by y = 3.1155x, with R^2^ = 0.9991. Based on the standard deviation of the blank response and the slope of this calibration curve, the estimated LOD and LOQ were 0.0654 and 0.1984 mM, respectively, according to the ICH Q2(R2) approach. These results demonstrate that the optimized electrode retained a linear analytical response at concentrations below the primary calibration range of 1.25–20 mM.

Within-electrode repeatability was evaluated by four consecutive measurements using the same electrode. At 5.5 mM glucose, the mean current density was 22.6218 ± 0.07083 μA cm^−2^, corresponding to an RSD of 0.31%. This low RSD indicates good repeatability of the glucose-dependent catalytic response. In glucose-free PBS, the mean background current density was 0.6596 ± 0.04625 μA cm^−2^, corresponding to an RSD of 7.01%. The higher relative variability of the blank response was mainly associated with its small mean current magnitude, whereas its absolute standard deviation remained low. The glucose-spiking curves showed prompt current changes within the 60 s interval between additions. Nevertheless, because the glucose solution was added manually without controlled convective mixing, the observed transient included both electrode-response and solution-mixing contributions. Therefore, an intrinsic numerical response time such as t90 could not be determined independently from the present measurements. The optimized sensor also exhibited a preferential glucose response compared with representative electroactive interferents, including ascorbic acid, uric acid, dopamine, and serotonin. In HPLM, the PVI-HEA-based electrode showed a more controlled electrochemical response than the conventional PVI-Os electrode, suggesting that the HEA-containing polymer composition may improve the behavior of the enzyme–mediator interface in a chemically complex medium. Nevertheless, HPLM is a chemically defined plasma-mimicking medium and not an actual human-derived plasma sample. Furthermore, glucose was externally added to the HPLM. Accordingly, these experiments demonstrate only preliminary matrix tolerance under controlled plasma-like conditions and do not establish the detection of endogenous glucose in unspiked human serum or plasma or constitute clinical validation.

The aqueous storage experiment provided additional information regarding the time-dependent performance of the complete enzyme electrode. The optimized electrode retained approximately 85–89% of its initial response during days 1–3 and subsequently retained 76.5%, 72.6%, and 68.6% on days 4, 5, and 6, respectively. Because the same electrodes were stored immersed in 1× PBS and measured once daily, these data represent aqueous storage stability under repeated measurement conditions rather than uninterrupted continuous operation. The gradual decrease indicates that, although measurable glucose-sensing activity was maintained for six days, further optimization is required for longer-term use.

The electrochemical retention of the immobilized mediator was evaluated by 250 consecutive CV cycles. The similar redox profiles and peak currents observed in the 1st and 250th cycles indicate that a substantial fraction of the electrochemically active Os mediator remained functional during repeated potential cycling. However, this experiment represents an indirect electrochemical assessment of mediator retention and not a direct quantitative leaching assay. Because the amount of Os released into the electrolyte was not chemically measured, the complete absence of mediator leaching cannot be concluded from the cycling data alone.

Continuous operational behavior was further evaluated by amperometric measurement for 12 h in 1× PBS containing 5.5 mM glucose. Following the initial transient period, the current density gradually decreased from approximately 22.3 to 18.8 μA cm^−2^, corresponding to an apparent current retention of 84.3%. Thus, most of the post-transient catalytic response was maintained during the 12 h measurement. Nevertheless, the observed 15.7% decrease should not be attributed exclusively to deterioration of the sensing layer. The experiment was conducted in a static batch solution without glucose replenishment, and continuous FAD-GDH-catalyzed oxidation may have progressively reduced the glucose concentration near the electrode surface and potentially in the bulk solution. Possible changes in enzyme activity, mediator distribution, mass transport, or the structural organization of the sensing layer may also have contributed. Because these factors were not independently measured, their individual contributions to the current decrease could not be distinguished.

Overall, the present results demonstrate that variation in the PVI-HEA copolymer composition provides an effective approach for tuning the electrochemical and glucose-sensing behavior of polymer-bound Os mediators. Among the tested compositions, PVI-HEA(3.5:1)-Os(dmo-bpy)_2_Cl_2_ exhibited the highest redox response and glucose sensitivity, an estimated LOD of 0.0654 mM, an LOQ of 0.1984 mM, and an RSD of 0.31% for repeated measurements at 5.5 mM glucose. The mediator retained its electrochemical activity during 250 CV cycles, while the complete enzyme electrode maintained 84.3% of its post-transient current response during 12 h of continuous measurement and 68.6% of its initial response after six days of aqueous storage. These findings support composition-dependent modification of the PVI-based polymer environment as a useful design strategy for FAD-GDH/Os redox-polymer glucose sensors. Further studies should include direct mediator-leaching analysis, controlled-flow or glucose-replenishment experiments, enhanced enzyme stabilization, protective and diffusion-controlling membrane layers, and validation using ethically approved human serum or plasma samples before practical CGM application.

## Data Availability

All data generated or analyzed during this study are included in this published article.

## Abbreviations

The following abbreviations are used in this manuscript:

CGM	Continuous glucose monitoring
GDH	Glucose dehydrogenase
GOx	Glucose oxidase
AA	Ascorbic acid
DA	Dopamine hydrochloride
UA	Uric acid
5-HT	Serotonin
HEA	2-Hydroxyethyl acrylate
PVI	1-Vinylimidazole
SPCEs	Screen-printed carbon electrodes
